# Computational study of the CO$_2$ hydrogenation to formic acid catalyzed by a P,P-bidentate copper(I) complex

**DOI:** 10.1007/s00894-026-06891-5

**Published:** 2026-08-13

**Authors:** Suelen A. Santos, Joel Leitão Nascimento, Tiago Vinícius Alves, Roberto Rivelino, Vitor H. Menezes da Silva

**Affiliations:** 1 Departamento de Físico-Química, Instituto de Química, Universidade Federal da Bahia, Rua Barão de Jeremoabo, 147, Salvador, 40170-115 BA Brazil; 2 Instituto de Física, Universidade Federal da Bahia, Salvador, 40210-340 BA Brazil; 3 Departamento de Química, Centro de Ciências Exatas, Universidade Estadual de Londrina, Londrina, 86050-482 PR Brazil

**Keywords:** Hydrogenation reaction, Organometallic compounds, Homogeneous catalysis, Capture of CO$_2$

## Abstract

**Context:**

Carbon dioxide (CO$_2$) hydrogenation is a promising route for converting CO$_2$ into value-added products. This work presents a computational investigation of CO$_2$ hydrogenation to formic acid catalyzed by the copper(I) complex [CuI(dtbpf)], previously reported as the P,P-bidentate 1,1’-bis(di-tert-butylphosphino)ferrocene (dtbpf)-ligated copper(I) complex. We employ electronic structure methods to characterize the catalytic cycle, highlighting the role of 1,8-diazabicyclo[5.4.0]undec-7-ene (DBU), a Lewis base, in H$_2$ heterolysis. Computational analyses revealed the interactions that govern Cu–H bond cleavage and Cu–O bond formation during the nucleophilic attack of the copper hydride on CO$_2$, as well as the role of DBU in the catalyst regeneration. Good correspondence was obtained between experimental and computed turnover frequency (TOF) values. Additionally, correlations involving the P-Cu-P bite angle and other geometric parameters were used to rationalize the stability of Cu-containing reaction intermediates, thereby providing insights into the electronic and steric effects of the complexes on the overall catalytic efficiency.

**Methods:**

Density functional theory (DFT) calculations at the B3LYP-D3/def2-TZVP//B3LYP-D3/def2-SVP (LanL2DZ for Cu) level were employed to examine the energy profile of the catalytic cycle. The energy span model (ESM) was used to quantify the turnover frequency (TOF) of the catalytic system. In addition, natural bond orbital (NBO), fuzzy bond order (FBO), and interaction region indicator (IRI) analyses were conducted to investigate the interactions within the complexes that govern the catalytic activity.

**Graphical abstract:**

**Figure:**
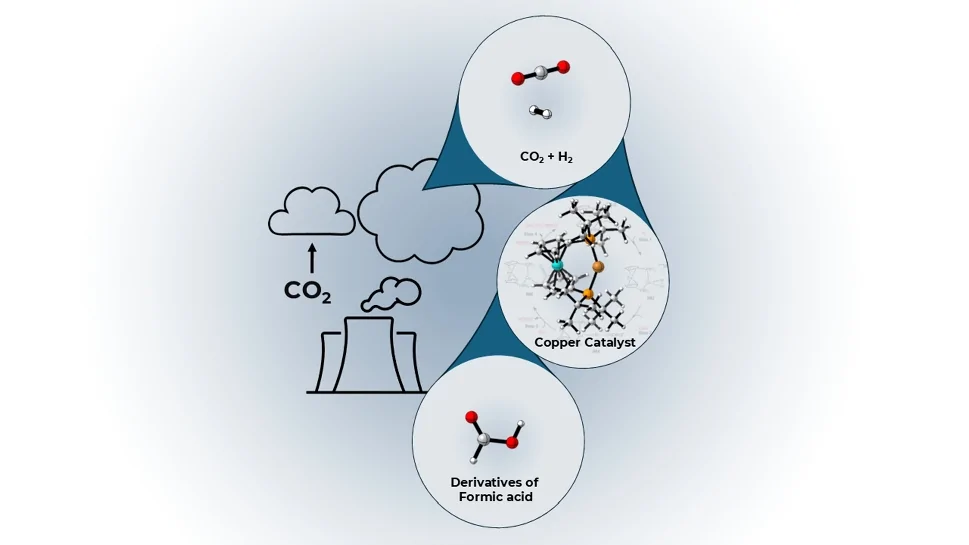

**Supplementary Information:**

The online version contains supplementary material available at https://doi.org/10.1007/s00894-026-06891-5.

## Introduction

Nowadays, the increasing concentration of carbon dioxide (CO$_2$) in the atmosphere represents one of the most critical global challenges [1, 2]. Based on the context of the carbon, capture, utilization, and storage (CCUS) [3], the catalytic hydrogenation of CO$_2$ emerges as a promising strategy to transform this gas into valuable chemicals, such as methanol [4, 5], methane [6], olefins [7], and formic acid (FA) [8]. Beyond contributing to carbon capture, these processes involving CO$_2$ also enable hydrogen storage, thereby integrating two crucial frontiers of sustainable chemistry [9]. Indeed, these CO$_2$ derivatives have attracted significant attention, particularly when homogeneous transition metal catalysts have been employed [10, 11]. In this sense, metal-mediated catalysis plays a pivotal role in achieving new efficient CO$_2$ transformations. CO2 is a thermodynamically stable molecule in the gas phase as its the standard free energy of formation (ΔG$^{o}_{f}$) is estimated to be −94.5 kcal/mol. As a consequence, CO$_2$ is also a well-known kinetically inert molecule under these conditions [12].

Specifically, FA is a compound with significant industrial relevance [13–15]. There is strong interest in developing alternative synthetic routes for FA production including its derivatives, although the hydrogenation of CO$_2$ to FA is thermodynamically unfavorable due to the endergonic reaction shown in Eq. 1 (ΔG$^{o}_{298}$ = 7.9 kcal/mol) [16].

1 $$\begin{aligned} \begin{array}{lcl} \text {CO}_2 + \text {H}_2\rightleftharpoons &   \text {HCO}_2\text {H} \end{array} \end{aligned}$$

2 $$\begin{aligned} \begin{array}{lcl} \text {CO}_2 + \text {H}_2 + \text {NH}_3\rightleftharpoons &   \text {HCO}_2^- + \text {NH}_4^+ \end{array} \end{aligned}$$

In this context, the addition of a Lewis base (LB) is the principal method employed to promote the conversion of FA to a more stable formate salt, as shown in Eq. 2 with a ΔG$^{o}_{298}$ estimated to be −2.3 kcal/mol [17]. For instance, this can be efficiently achieved using 1,8-diazabicyclo[5.4.0]undec-7-ene as the Lewis base (LB-DBU), which reacts rapidly to yield the product via the [HCOO]$^{-}$ [H-DBU]$^{+}$ ion-pair [18, 19]. The reaction between DBU and formic acid shifts the chemical equilibrium towards formate formation, thus enhancing the thermodynamic driving force for the reaction to progress, in accordance with Le Chatelier’s principle [20]. In this context, Lewis bases (LBs), such as DBU, facilitate the formation of the formate/formic acid product [21].

Numerous studies have focused on the development of catalysts based on non-noble transition metals, aiming to achieve high efficiency, selectivity, economic viability, and environmental sustainability [22–25]. Recent advances in homogeneous catalytic hydrogenation of CO$_2$ using 3*d* transition metal complexes have expanded significantly [26–28]. Notably, Wedal et al. described an unusual PNP-pincer iron complex with a remarkable hemilabile donor that effectively promotes CO$_2$ hydrogenation to FA. This work highlights the importance of incorporating an auxiliary donor ligand as a crucial strategy to optimize catalytic performance in CO$_2$ hydrogenation to FA [29]. In addition, Klankermayer et al. presented an active nickel(II) salt in combination with a multidentate ligand as a catalyst, achieving an exceptional TON of approximately $4.65 \times 10^6$ (using $\text {0.002 }\mu \text {mol}$ of catalyst) [30]. In this direction, Beller et al. demonstrated that Mn–PNP complexes achieve CO$_2$ hydrogenation reversibly with high efficiency. This reaction system directly hydrogenated CO$_2$ to formate with a record TON of 2,000,000 and, conversely, generated H$_2$ from FA with a TON of 600,000 [31].

Copper-based organometallics and coordination complexes have shown promising catalytic activity in CO$_2$ hydrogenation [26, 32]. The critical steps of the catalytic cycle have been related to the formation of copper hydrides to promote the CO$_2$ reduction [33, 34]. However, key challenges remain, particularly the direct experimental identification of reaction intermediates, which limits the ability to gain deeper mechanistic insights [34, 35]. Typically, the activation of H$_2$ by a metal complex involves the formation of an intermediate known as a σ-complex, in which the H$_2$ molecule binds to the copper center before H–H bond cleavage takes place [36]. However, these combined processes are very difficult to achieve experimentally because H$_2$ is a small and labile ligand and may not remain bound to the copper center long enough for the formation of hydride species. In addition, the bond dissociation energy (BDE) of the H–H bond is very high, approximately 104 kcal/mol [37, 38], explaining the crucial role of Lewis bases (LBs) in H$_2$ activation. In this context, Chaudhary et al. reported copper(I) complexes containing the 1,1’-bis(di-tert-butylphosphino)ferrocene ligand to promote the catalytic hydrogenation of CO$_2$ to formate in the presence of DBU [39]. Specifically, their experimental strategy involved conducting reactions under ambient pressure and at room temperature, achieving CO$_2$ hydrogenation catalysis to generate the formate salt HCO$_{2}^{-}$ with turnover number (TON) values ranging from 326 to $1.065 \times 10^5$ after 12 to 48 h of reaction at room temperature. Furthermore, the reaction mechanism was investigated using NMR spectroscopy, providing valuable insights that motivated us to carry out a computational study to further expand the understanding of the catalytic cycle.

Taken together, the challenges underscore the critical importance of theoretical and computational studies to elucidate the catalytic cycle mechanisms related to CO$_2$ hydrogenation to formic acid in the presence of an external LBs as additives. In this context, the goal of this study is to employ density functional theory (DFT) calculations to provide a detailed investigation of the catalytic cycle related to the conversion of CO$_2$ into formate catalyzed by the [Cu(dtbpf)]$^+$ complex [39]. To this end, the reaction Gibbs free energies were computed and further analyzed using the energy span model (ESM). Furthermore, natural bond orbital (NBO), fuzzy bond order (FBO), and interaction region indicator (IRI) analyses were conducted to investigate the interactions that govern the stability of key reaction intermediates. In addition, energetic and geometrical parameters were correlated to elucidate the catalytic efficiency. Specifically, the P-Cu-P bite angle is particularly important for understanding the electronic and steric features of the complexes. We expect that these computational findings will shed light on key mechanistic features of CO$_2$ hydrogenation to formic acid catalyzed by the copper(I) complex [CuI(dtbpf)].

## Computational methods

All electronic structure calculations were performed using the Gaussian 09 program [40]. The geometry optimization and frequency calculations were carried out at the B3LYP density functional, combined with the D3 empirical dispersion correction [41, 42] using an “ultrafine” integration grid. For these calculations, the def2-SVP basis set was employed for all main-group atoms [43], except for copper, for which the LanL2DZ basis set was used [44, 45] (this specification is denoted as BS1). This level of theory has been successfully applied to predict the experimental TOF data from the free energy barrier computed for the TOF-determining step (*vide infra*) [39]. The solvation effects were included using the implicit polarizable continuum model (PCM) [46]; the integral equation formalism variant (IEFPCM) was employed as the default SCRF method, using the parameters of DMF as the solvent (DMF was the preferred solvent in the experimental work) [39].

All stationary points were further characterized by vibrational analysis: transition states exhibited a single imaginary frequency corresponding to the reaction coordinate. Due to a flat potential energy surface (PES), the connectivity among minima and transition states was verified by slightly distorting the transition state structures along the imaginary frequency vector, followed by directed geometry optimizations using calcfc and a reduced “maxstep=10.” Free energies were evaluated using a direct approach from single-point (SP) calculations at a higher basis-set level (B3LYP-D3/def2-TZVP//B3LYP-D3/def2-SVP (LanL2DZ for Cu)) [47]. Specifically, the final $G_\textrm{sol}$ values were determined by combining the thermal corrections (*T* = 298.15 K and *P* = 1 atm) computed in the DMF at the BS1 level (B3LYP-D3/def2-SVP and LanL2DZ for Cu) with the electronic energies in solution obtained from the single-point SCRF calculations at the BS2 level (B3LYP-D3/def2-TZVP). Additionally, the standard-state concentration correction ($\Delta G^{\circ }_\mathrm{1 atm \rightarrow 1 M} = 1.89$ kcal mol$^{-1}$) was consistently applied to all reaction components, as described by Eq. 3:

3 $$\begin{aligned} G_\textrm{sol} = E^\textrm{SP}_\textrm{BS2} + G^{T,P}_\textrm{corr, BS1} + \Delta G^{\circ }_{\text {1 atm} \rightarrow \text {1 M}} \end{aligned}$$

Energy span values were computed according to the energy span model (ESM) [48–50]. This model quantifies the kinetic feasibility of a catalytic system by identifying the pair of intermediate and transition state that most significantly impacts the reaction rate (TOF of catalytic cycle), as obtained from the free energy profile computed from the DFT calculations. In this context, the energetic span, δE, is defined as the energy difference between the TOF-determining intermediate (TDI) and the TOF-determining transition state (TDTS), as detailed in Eq. 4.

4 $$\begin{aligned} \delta E = \left\{ \begin{array}{ll} T_i - I_j &  \text {if } i > j \quad \text {(a)} \\ T_i - I_j + \Delta G &  \text {if } i \le j \quad \text {(b)} \end{array}\right.  \end{aligned}$$

We employed natural bond orbital (NBO) analysis to understand how the P–Cu–P bite angle affects the electronic characteristics of the species involved in selected mechanistic pathways [51]. The second-order perturbation stabilization energies, $E^{(2)}$, were evaluated at the B3LYP-D3/BS1 level of theory. Additionally, interaction region indicator (IRI) [52] and fuzzy bond order (FBO) [53, 54] analyses were performed using Multiwfn [55, 56] to investigate the interactions that govern the stability of the key reaction intermediates.

## Results and discussion

### Catalytic cycle steps

**Fig. 1:**
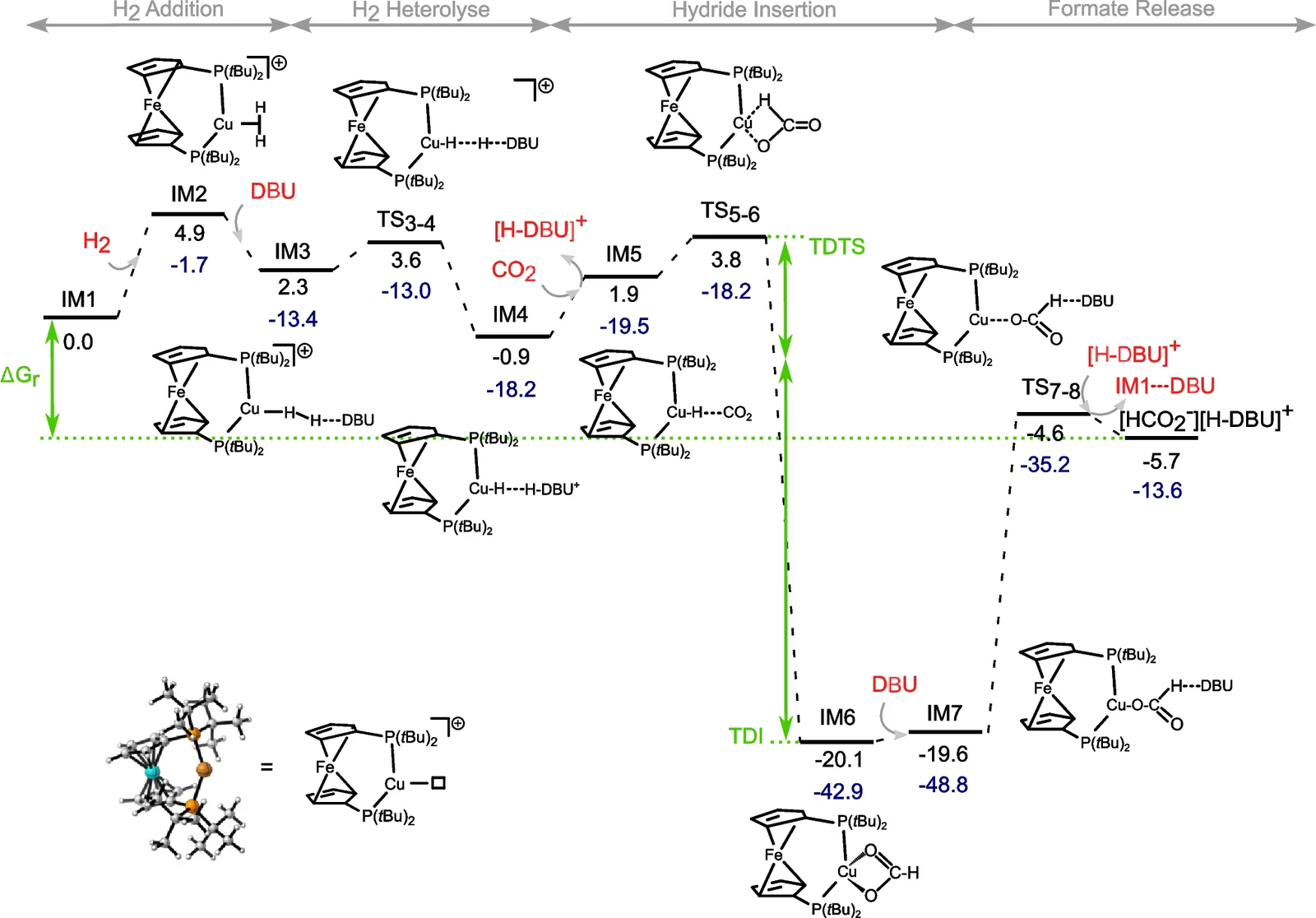
Free energy profile of catalytic cycle of CO$_2$ hydrogenation to formic acid in the presence of DBU catalyzed by [Cu(dtbpf)]$^+$. Relative energies were computed relative to catalyst [Cu(dtbpf)]$^+$ (**IM1**). The ΔG values are reported in black and ΔH values reported in blue. All energy values are in kcal/mol and computed at 298.15 K and 1 M

The corresponding reaction free energy profile for the catalytic cycle of CO$_2$ hydrogenation to formic acid in the presence of DBU as the Lewis base catalyzed by [Cu(dtbpf)]$^+$ is shown in Fig. 1 (the reaction enthalpy is also reported). The profile comprises four main reaction steps: (1) H$_2$ coordinates to the [Cu(dtbpf)]$^+$ complex giving the Cu dihydrogen σ-complex; (2) H$_2$ activation occurs through heterolytic bond cleavage assisted by DBU, leading to the formation of the Cu hydride species; (3) the nucleophilic attack of the Cu hydride on CO$_2$ following the hydride insertion into the CO$_2$, which forms the Cu-formate intermediate; (4) the formate (or formic acid) is released through ion-pair [HCOO]$^{-}$ [H-DBU]$^{+}$, thus regenerating the active catalyst [Cu(dtbpf)]$^+$ and restarting the catalytic cycle.

Analyzing first the activation of the catalyst, the cationic [Cu(dtbpf)]$^+$ complex is formed by the dissociation of iodide (I$^-$) from the initial neutral [CuI(dtbpf)] complex, which is feasible in a polar reaction medium (DMF solvent). In this sense, the active catalyst [Cu(dtbpf)]$^+$ (**IM1**) first reacts with molecular hydrogen (H$_2$) to form the corresponding σ-complex adduct **IM2**. Scrutinizing **IM2**, this intermediate is a dihydrogen complex containing intact H$_2$ as a ligand. The formation of **IM2** is slightly endergonic (4.9 kcal/mol).

The next step is H$_2$ activation. As DBU was the most effective base for the CO$_2$ hydrogenation reaction [39], we decided to use the Lewis base DBU in the reaction model. As a result, the adduct DBU⋯[CuH$_2$(dtbpf)]$^+$ (**IM3**) was computed to be 2.6 kcal/mol more stable than **IM2**, thus indicating that DBU starts to interact with the H$_2$ of **IM3** complex. Indeed, DFT calculations reveal that DBU promotes a feasible heterolysis of H$_2$ via the transition state ${\textbf {TS}}_{3-4}$, with the product of this elementary step being the copper hydride **IM4**. Indeed, the free energy barrier for the H-H bond cleavage is computed to be exceptionally low, only 1.3 kcal/mol. To illustrate the important role of DBU in H$_2$ activation, the heterolytic cleavage of H$_2$ to form $\text {H}^-$ and $\text {H}^+$, in the absence of Lewis base, it was observed to be highly thermodynamically demanding, requiring a ΔG of formation of +76.0 kcal/mol in acetonitrile solvent [57]. The product of the H$_2$ activation is the copper hydride complex **IM4**.

In the third catalytic step, the nucleophilic attack of the copper hydride takes place, i.e., a hydride insertion reaction into CO$_2$. Our DFT calculations indicate that CO$_2$ interacts intermolecularly, forming the CO$_2$ ⋯[CuH(dtbpf)]$^+$ complex (**IM5**). The hydride is a strong nucleophile (as the hydrogen atom is more electronegative than the Cu atom), and it promptly reacts with the weakly electrophilic carbon atom of CO$_2$, forming a new C–H bond. Likewise to the previous step, the insertion of the hydride into CO$_2$ is rapid and occurs through a concerted transition state ${\textbf {TS}}_{5-6}$, with a free energy barrier of only 1.9 kcal/mol. This reaction step is strongly exergonic, demonstrating the remarkable stability of **IM6** (ΔG = −20.1 kcal/mol).

The last stage has been evidenced by the calculations to involve the formation of the formate complex [CuCO$_2$H(dtbpf)]$^+$, named as **IM6**, which possesses a $\kappa ^2$-coordinated binding mode. In contrast, previous experimental reports on this complex formation have suggested a $\kappa ^1$-coordination mode [39, 58]. It is important to highlight that both coordination modes were successfully optimized as stable local minima on the PES (see Fig. S3). Our thermodynamic analysis reveals that the $\kappa ^1$ and $\kappa ^2$ isomers are isoenergetic, with the $\kappa ^1$ form being favored by 0.1 kcal/mol. Thus, the two coordination modes are expected to coexist in a dynamic equilibrium in solution. However, during our systematic investigation of the reaction pathways starting from the transition state, the optimization steps consistently guided the system toward the bidentate $\kappa ^2$ form being the kinetically relevant species in our proposed mechanism. The two newly formed Cu–O coordinate covalent bonds were computed to be 2.27 Å, confirming good accordance with the average distances of typical Cu–O bonds in copper coordination complexes reported in X-ray crystallographic studies. Furthermore, the resulting formate ligand interacts through one of its oxygen atoms with the conjugate acid of DBU (H$^+$DBU) previously formed. Indeed, the Cu–O bond in **IM7** is elongated by 0.20 Å, possibly indicating partial oxygen decoordination from the copper center due to this ion-pair interaction. Finally, the formate (or formic acid) is released as [HCOO$^-$]H[DBU]$^+$ ion-pair, and the active catalyst is regenerated as the van der Waals (vdW) complex **IM1** ⋯ **DBU**, thereby restarting the catalytic cycle.

### Theoretical versus experimental TOF

To evaluate the catalytic efficiency of the system, we performed a kinetic investigation employing the energetic span model (ESM) proposed by Kozuch and Shaik [48, 59] to analyze the experimental mechanistic findings [39]. The ESM indicates that there is no single rate-limiting step; instead, the kinetics is governed by determining states defined as the TDTS (TOF-determining transition state) and the TDI (TOF-determining intermediate). An accurate identification of the TDI and TDTS requires analyzing all possible combinations of intermediates and transition states throughout the catalytic cycle.

As illustrated in Fig. S2, the free energy profile was rationalized using the ESM model. It is clear that the TDI of the catalytic cycle is the formate complex [CuCO$$_2$$H(dtbpf)]$$^+$$ (**IM6**). However, the choice of the TDTS should be made carefully. As detailed in Eq. 4, when the TDI appears before the transition states $${\textbf {TS}}_{3-4}$$ and $${\textbf {TS}}_{5-6}$$, the reaction free energy ($$\Delta G_r$$) must be added to estimate $\delta E^{\prime }$. When $${\textbf {TS}}_{5-6}$$ is selected as the TDTS, according to Eq. 4, the $\delta E^{\prime }$ value is computed to be 18.2 kcal/mol, considering that the TDTS, TDI, and $$\Delta G_r$$ were estimated to be 3.8, −20.1, and 5.7 kcal/mol, respectively. Notably, the energetic span obtained when TS$$_{3-4}$$ is considered the TDTS differs by only 0.2 kcal/mol (18.0 kcal/mol), indicating that both transition states contribute comparably to the catalytic turnover and suggesting a shared TOF control scenario within the expected accuracy of the DFT methodology. Both values are in good agreement with the experimental activation free energy ($$\Delta G^\ddagger $$) estimated using the Eyring equation (20.3 kcal/mol), as well as with the experimentally determined TOF based on the experimental data report: a maximum turnover number (TON) of 326, obtained using a base/Cu catalyst ratio of 500 in DMF at 25 $$^{\circ }$$C under 1 atm of CO$$_2$$/H$$_2$$ for 12 h [39]. In this sense, as $${\textbf {TS}}_{3-4}$$ and $${\textbf {TS}}_{5-6}$$ provide practically equivalent δE values (18.0 and 18.2 kcal/mol), it is not possible, based on the DFT calculations, to single out a unique TDTS for the catalytic cycle. In other words, both the H$$_2$$ activation and the hydride insertion into CO$$_2$$ steps contribute significantly to the overall catalytic efficiency.

It is worth pointing out some considerations of ESM model obtained from the computed catalytic cycle and the experimental findings. Chaudhary et al. investigating the reaction mechanism by NMR spectroscopy [39] noticed that the association/deprotonation of H$_2$ to form the copper hydride is apparently transient. The authors observed the copper hydride over the course of the reaction, but it possibly reacts quickly and does not accumulate. Specifically, by $^1$H NMR analysis, the copper hydride was identified with a well-resolved chemical shift (a δ signal in the region from -0.11 to -0.17 ppm) showing the Cu–H presence. However, this signal appears only for a short period of the reaction time, reinforcing its high reactivity and the kinetic feasibility of the H$_2$ activation process. The ESM model follows the Curtin–Hammett principle, i.e., the intermediates are in fast pre-equilibrium and the TOF is determined solely by the energy difference between the TDI and the TDTS. Therefore, the complete elucidation of copper hydride isolation would require microkinetic models, which are beyond the scope of the present computational study.

Furthermore, experimental findings have suggested the hydride formation as the rate-limiting step of the reaction. In this context, the superior catalytic performance provided by DBU is associated with its ability to promote the heterolytic splitting of H$$_2$$ within the copper coordination sphere, thereby facilitating the formation of the copper hydride complex. Moreover, DBU enables the facile formation of a thermodynamically stable ion pair as ligand in the Cu coordination sphere, [HCOO]$$^{-}$$ [H-DBU]$$^{+}$$, which facilitates the formate release and catalyst regeneration. These experimental observations are in good agreement with the kinetic analyses based on the ESM model, indicating the possibility of $${\textbf {TS}}_{3-4}$$ acting as the TDTS, as well as highlighting the importance of **IM7** possessing [HCOO]$$^{-}$$ [H-DBU]$$^{+}$$ as ligand. For instance, when **IM7** is considered the TDI, $\delta E^{\prime }$ values of 17.7 or 17.5 kcal/mol were obtained, in concordance with the participation of **IM7** in determining the catalytic efficiency.

### Analysis of the key reaction intermediates of the catalytic cycle

**Fig. 2:**
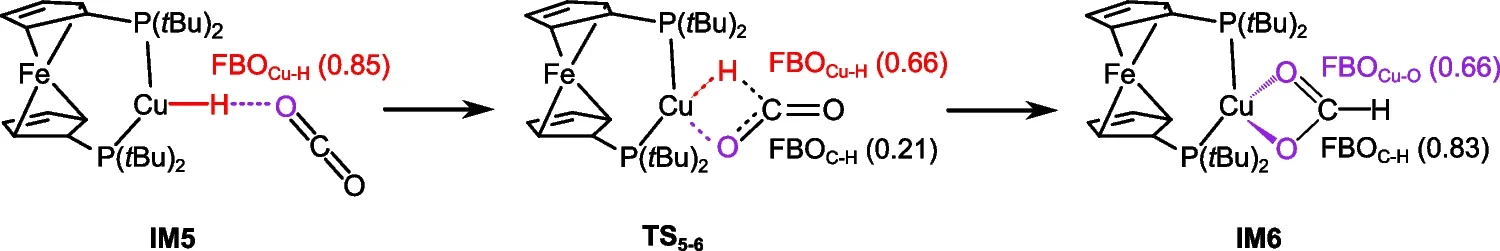
The copper hydride insertion reaction. Fuzzy bond orders (FBO) are highlighted in the selected bonds. The different FBO colors correspond to red, purple, and black, representing the Cu–H, Cu–O, and C–H bonds, respectively

In order to provide a more detailed discussion of the catalytic features of the reaction, we carried out two complementary computational analyses to investigate the interactions involved in the formation of the formate/formic acid product. First, FBO (fuzzy bond order) values were obtained for the Cu complexes involved in the insertion of the copper hydride into CO$_2$ (Fig. 2).

FBO analysis provides a quantitative measure of chemical bond strength in each complex [53, 54], thereby allowing us to track bond breaking and bond formation throughout the reaction process. Indeed, for the Cu–H bond in the reactant complex, an FBO value of 0.85 was computed, revealing a well-established hydride character. On the other hand, in the transition state **TS** $_{5-6}$ , the FBO value for the Cu–H bond decreases to 0.66, while the emerging C–H bond remains weak (FBO = 0.21), indicating that hydride transfer is underway. Finally, for intermediate **IM6**, the Cu–H bond is no longer evident (FBO = 0.00), whereas the Cu–O bond becomes significantly stronger (FBO = 0.66), concomitantly with formation of the C–H bond (FBO = 0.83), thus evidencing generation of the formate product. These newly formed strong covalent bonds help explain the pronounced exothermicity computed for insertion of the copper hydride into CO$_2$, as shown in the free energy profile in Fig. 1. In addition, we performed an IRI (interaction region indicator) analysis, a bond analysis tool that enables visualization and classification of different types of covalent and non-covalent interactions present in a molecular structure [52]. Figure S4 displays the density-gradient profile associated with the copper hydride insertion into CO$_2$. It can be seen that the copper hydride complex **IM5** is initially stabilized by van der Waals interactions involving CO$_2$ and the Cu–H bond, indicating the high polarizability of the hydride moiety. However, these interactions do not show preferential directionality, as would be expected for hydrogen bonding. Overall, the results indicate the presence of significant non-covalent interactions that govern the stability of copper hydride complex **IM5** involving the ligands in the Cu coordination sphere.

**Fig. 3:**
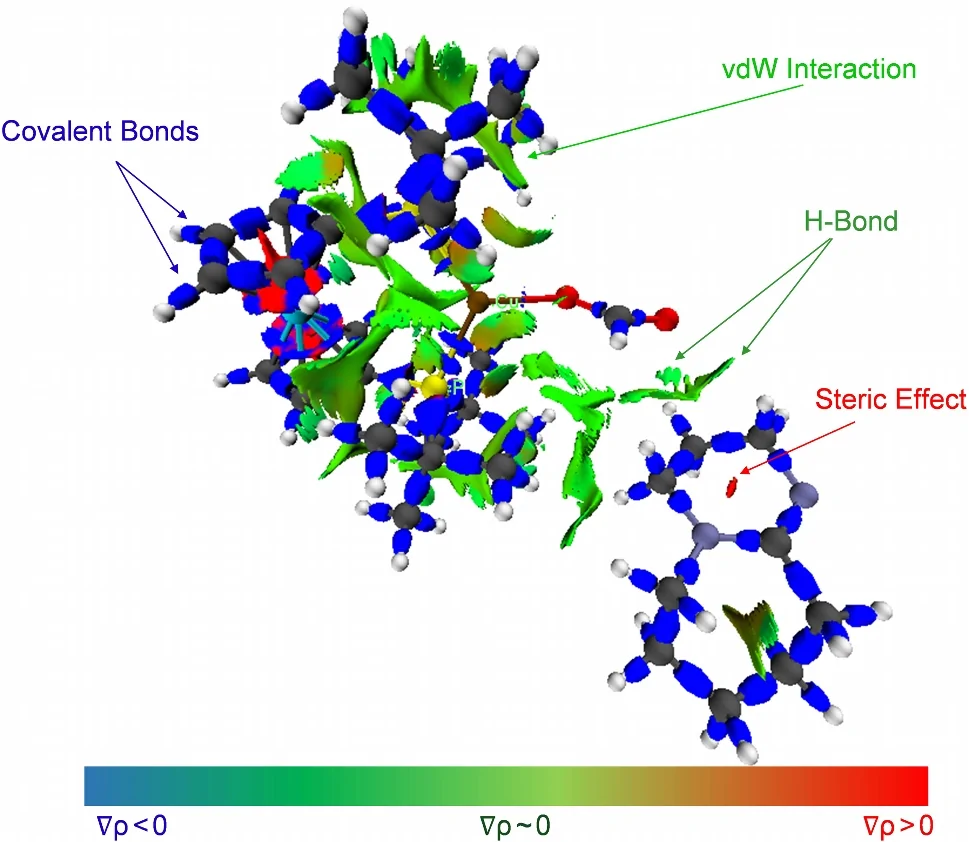
IRI (interaction region indicator) analysis of **IM7**. The density-gradient profile indicates the strength and nature of the interactions, where blue corresponds to strong attractive interactions, while red denotes repulsive interactions

Another key feature of the catalytic activity revealed by the calculations concerns the stability of the ion pair [HCOOH$^+$][DBU], which facilitates formate/formic acid release and catalyst regeneration. In this scenario, DBU plays a pivotal role as Lewis base, as indicated by the IRI (interaction region indicator) analysis of intermediate **IM7** (see details in Fig. 3).

It is possible to observe a significant region of non-covalent interaction between DBU and the product species (formate/formic acid), convincingly indicating the presence of stabilizing interactions in the complex **IM7**. These interactions help promote formation of the **IM7** and, consequently, drives the subsequent regeneration of the cationic Cu catalyst.

**Fig. 4:**
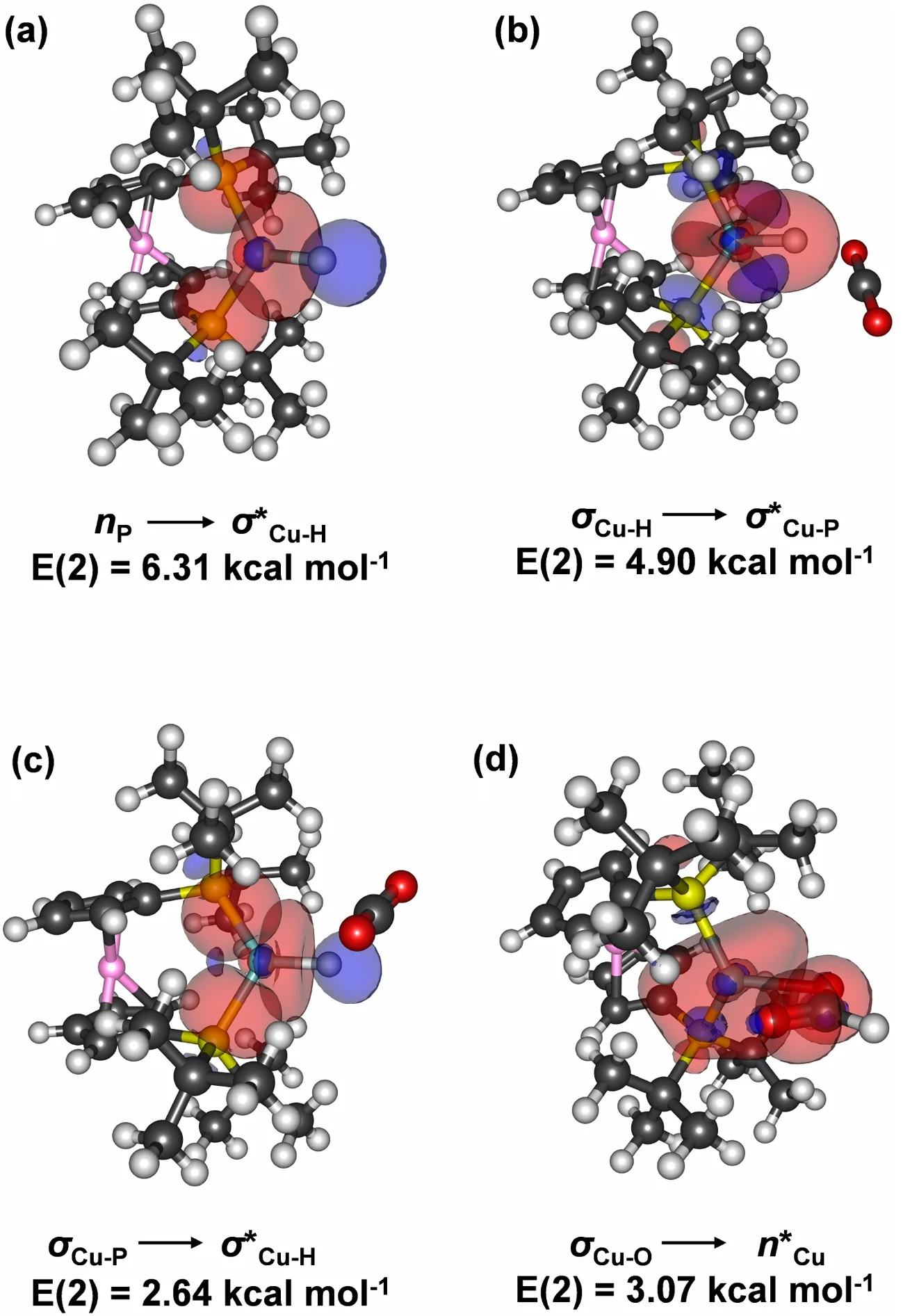
Orbital interactions involved in the hyperconjugation effect of the hydride insertion obtained from NBO analysis. The second-order perturbation stabilization energy, *E*(2), is in kcal/mol. Arrows indicate the donor → acceptor direction. ***a*** Copper(I) hydride, ***b*** **IM5**, ***c*** **TS** $_{5-6}$ , and ***d*** **IM6**

At the final stage of this computational investigation, we analyzed the geometrical parameters of key reaction intermediates in order to gain further understanding of the catalytic activity of these P,P-bidentate complexes (see details in Tab. S2). Specifically, we investigated the influence of geometrical parameters on the stability of intermediates and transition states throughout the catalytic cycle for CO$_2$ hydrogenation catalyzed by the Cu(I) diphosphine active catalyst [Cu(dtbpf)]$^+$ (**IM1**). The bite angle is defined by the P–Cu–P geometry formed by the two phosphine ligands coordinated to the copper center, and its variation can directly influence orbital overlap and the efficiency of the metal–ligand interaction through hyperconjugative effects involving the orbitals at the catalytic site [60–62]. It has previously been shown that changes in the P–Cu–P bite angle enhance the interaction between the phosphorus lone pair ($n_\textrm{P}$) and the $\sigma ^{\text {Cu}-\text {H}}$ orbital, resulting in a more pronounced hydride character [60]. To further illustrate this effect, Fig. 4 shows the NBOs of the key atoms defining the bite angle together with the substrate coordinated to the Cu center during hydride insertion into CO$_2$. The hyperconjugative effect can be clearly observed during the hydride transfer process through the $n_\textrm{P} \rightarrow \sigma ^*_{\text {Cu}-\text {H}}$ orbital interaction, consistent with the NBO analysis reported by Gathy et al. [60]. Due to the well-known limitations of NBO analyses involving transition metal centers [63–65], the associated E(2) stabilization energies should be discussed only qualitatively to support the role of bite angle on the catalytic activity.

**Fig. 5:**
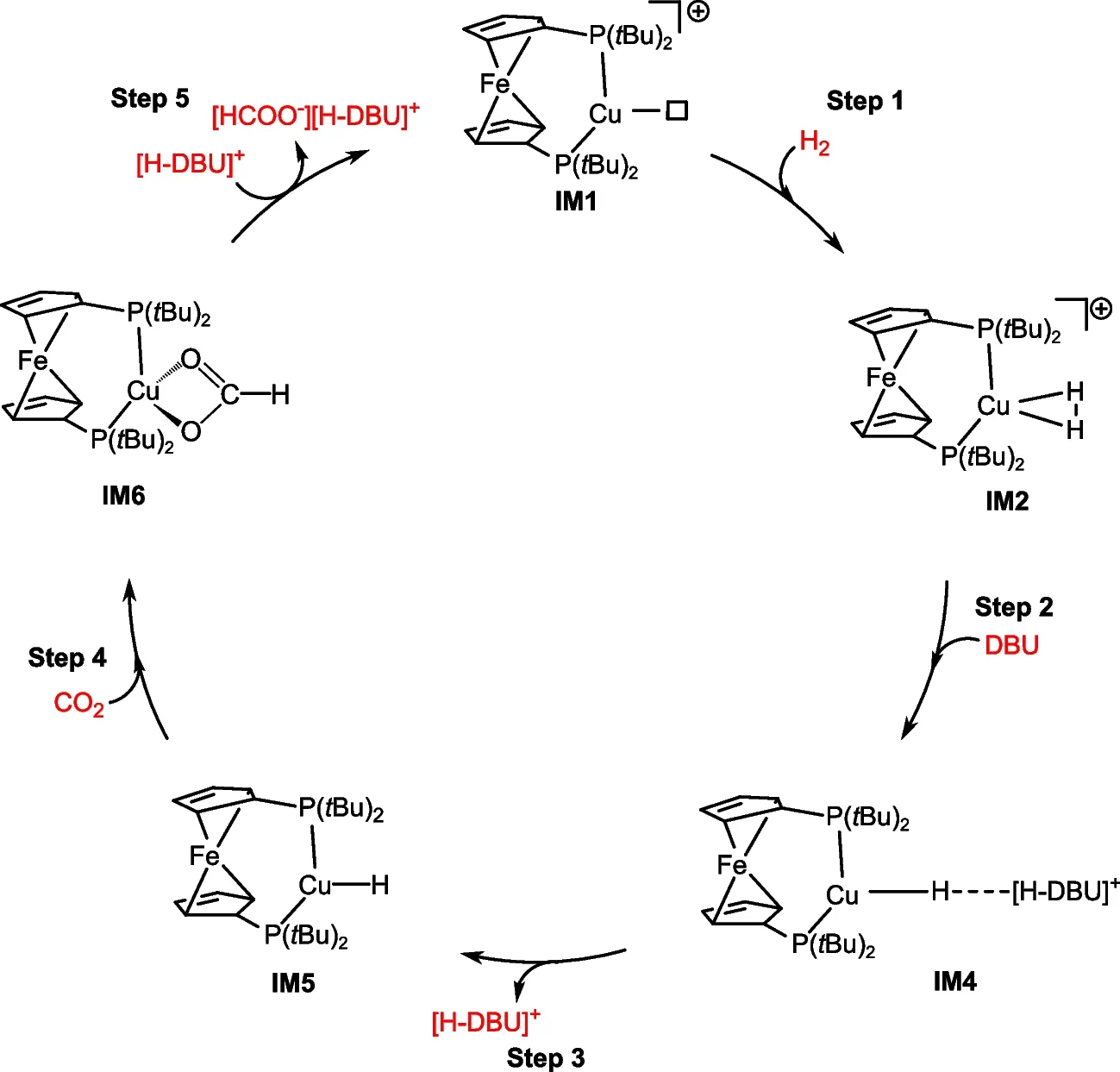
Proposed mechanism for CO$_2$ hydrogenation catalyzed by the [Cu(dtbpf)]$^+$ complex

The bite angle of the active catalyst [Cu(dtbpf)]$^+$ (**IM1**) was computed to be 137.2$^{\circ }$, which is in reasonable agreement with the P–Cu–P bite angle of 120.070(19)$^{\circ }$ obtained from X-ray crystallographic data for the initial catalyst [CuI(dtbpf)] [66]. In Table S2, we report the $\Delta \theta$ values of the P–Cu–P bite angle, as well as the phosphine–Cu bond distances associated with the P,P-bidentate ligand for the different reaction intermediates. Throughout the progression of the reaction mechanism, an increasing geometric distortion is observed from **IM1** to **IM5**, with the $\Delta \theta _{\text {P}-\text {Cu}-\text {P}}$ values becoming more negative; that is, the P–Cu–P bite angle decreases, reflecting the progressive saturation of the Cu(I) center during H$_2$ cleavage and formation of the hydride intermediate. Furthermore, the phosphine–Cu bond distances elongate during these steps, which also supports increased saturation at the Cu(I) center with the formation of the formate in the coordination sphere. Indeed, these geometrical changes indicate the active involvement of the metal and ligand center during H$_2$ activation and generation of the copper(I) hydride complex. The largest deviations in bite angle and phosphine–Cu bond distances occur for **IM4**, **IM5**, and **TS** $_{5-6}$ , with variations greater than 20$^{\circ }$ and 0.08 Å, respectively, indicating substantial geometrical change relative to the initial catalyst. At this stage of the catalytic cycle, particularly during insertion of the hydride complex into CO$_2$, a strongly exergonic pathway is observed, possibly due to saturation of the Cu(I) complexes. In addition, this specific bite angle likely favors back-donation from the phosphine lone pairs to the metal center, which may facilitate CO$_2$ activation, i.e., hydride insertion into CO$_2$. On the other hand, during the conversion of **IM6** to **IM7**, Table S2 shows an increase in the P–Cu–P bite angle and a slight shortening of the phosphine–Cu bond distances. However, the free energy changes for the formation of these species remain strongly exergonic (more negative ΔG values). This suggests that, at this stage, the steric features of the P,P-bidentate ligand within the coordination sphere play a more dominant role than geometry in stabilizing the intermediates, particularly through the formation of stabilizing non-covalent interactions between DBU and formate, as revealed by the IRI analysis (see Fig. 3), as well as the newly formed strong covalent bonds with Cu(I) ion revelead by the FBO analysis (see Fig. 2). Finally, in transition state **TS** $_{7-8}$ , the $\Delta \theta$ value returns to approximately $0^{\circ }$, indicating restoration of the initial geometry of the active catalyst and thereby enabling catalyst regeneration.

A computational view of the carbon dioxide hydrogenation mechanism obtained through DFT calculations is summarized in Fig. 5.

The catalytic cycle begins with the coordination of H$_2$ to the [Cu(dtbpf)]$^+$ complex (**Step 1**), forming a copper dihydrogen σ-complex. Subsequently, the Cu(I) center promotes the heterolytic cleavage of the H$_2$ bond assisted by DBU (**Step 2**), leading to the formation of the copper hydride species, which intercepts CO$_2$ intermolecularly to form **IM5** (**Step 3**). This key complex sets the stage for the subsequent CO$_2$ hydrogenation process. Insertion of the Cu–H bond into CO$_2$ then takes place, generating a copper-formate intermediate (**Step 4**). In the final stage of the cycle (**Step 5**), formic acid is released from the coordination sphere of the Cu(I) complex, thereby regenerating the active catalyst [Cu(dtbpf)]$^+$ and restarting the catalytic cycle.

## Conclusions

The present computational study investigated the catalytic cycle of the P,P-bidentate [CuI(dtbpf)] complex in CO$_2$ activation, highlighting its ability to promote the catalytic hydrogenation of CO$_2$ resulting in formate in the presence of DBU. Analysis of the catalytic cycle revealed that the initial stages of the reaction are turnover frequency (TOF)-determining. In particular, the heterolytic cleavage of the H$_2$ molecule and the subsequent insertion of the Cu(I) hydride into CO$_2$ are the key steps that govern the overall rate, in good agreement with the experimental data. However, considering the intrinsic accuracy limits of DFT calculations, it was not possible to single out which of these steps is the TOF-determining process, despite the excellent correspondence between experimental and computed TOF values. We also carried out complementary computational bonding analyses of key Cu-containing intermediates in order to identify the electronic and steric features responsible for the catalytic activity. Furthermore, the influence of the P–Cu–P bite angle, together with other geometrical parameters, was correlated with the stability of intermediates and transition states, revealing the important role of the P,P-bidentate 1,1’-bis(di-tert-butylphosphino)ferrocene ligand in promoting the formation of key Cu(I) reaction intermediates. In general, a specific bite angle associated with saturation of the Cu(I) complex favors back-donation from the phosphine lone pairs to the metal center, which may kinetically and thermodynamically facilitate hydride insertion into CO$_2$. On the other hand, during catalyst regeneration, the bite angle resembles that of the initial catalyst. At this stage, the steric features of the Cu(I) complexes, particularly those promoted by the presence of DBU, appear to be essential for the catalytic feasibility. These computational findings reinforce the catalytic potential of copper-based P,P-bidentate complexes with large bite angles as efficient homogeneous catalysts for CO$_2$ hydrogenation, while providing valuable insights for the rational design of more effective and sustainable catalytic systems.

## Supplementary information

Electronic Support information contains further computational data and Cartesian coordinates of all stationary points obtained with their respective SCF energies.

## Supplementary Information

Below is the link to the electronic supplementary material.

Supplementary file 1 (pdf 8512 KB)

## Data Availability

No datasets were generated or analysed during the current study.
