# Supplementary material for: Computational study of the CO2 hydrogenation to formic acid catalyzed by a P,P-bidentate copper(I) complex
Source: J Mol Model. 2026 Aug 13;32(9):310. doi: 10.1007/s00894-026-06891-5 (PMC13469215; doi:10.1007/s00894-026-06891-5)
Supplement: Supplementary file 1 [file 894_2026_6891_MOESM1_ESM.pdf]

Electronic Supplementary Information (ESI)

Computational Study of the CO_2 Hydrogenation to Formic Acid Catalyzed by a P,P-Bidentate Copper(I) Complex

Suelen A. Santos,[1] Joel Leitão Nascimento,[1], Tiago V. Alves[3*], Roberto Rivelino[2*], Vitor H. Menezes da Silva[3*]

[1] *Departamento de Físico-Química, Instituto de Química, Universidade Federal da Bahia, Rua Barão de Jeremoabo, 147, Salvador, Bahia, 40170-115, Brazil*

[2] *Instituto de Física, Universidade Federal da Bahia, Salvador, Bahia, 40210-340 , Brazil.*

[3] *Departamento de Química, Centro de Ciencias Exatas, Universidade Estadual de Londrina, Londrina, Paraná, 86050-482 , Brazil.*

*E-mail address: *tiago.alves@uel.br, *rivelino@ufba.br, *vmenezesdasilva@uel.br

Sections

S1. Global Reaction S3

S2. Multiplicity S3

S3. Pairs determining Step (TDI – TDTS) S4

S4. Coordination Mode S4

S5. IRI and FBO Analysis S5

S6. Bite Angle Analysis S6

S7. Cartesian Coordinates S7

Tables

S1. Relative energies of the $[Cu(dtbpf)]^+$ complex in singlet and triplet states . S3

S2. Influence of key bond distances and bite angle on the stability of intermediates and transition states in the catalytic cycle S6

Figures

S1. Global reaction between formic acid and LB-DBU S3

S2. Assessment of potential TDTS and TDI Pairings. Relative free energies ΔG (in black) in kcal.mol^{-1} at 298.15K and 1atm S4

S3. Eletronic structure of κ^1 and κ^2 isomers in DMF S4

S4. Analysis of IRI (Interaction Region Indicator) and FBO (Fuzzy Bond Order) of the interactions involved in formate formation. S5

S1. Global Reaction

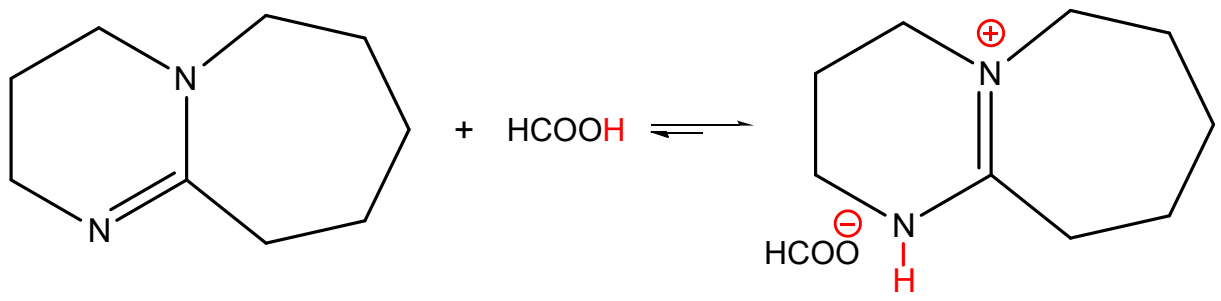

Fig. S1.: Global reaction between formic acid and LB-DBU

S2. Multiplicity

Table S1.: Relative energies of the $[Cu(dtbpf)]^+$ complex in singlet and triplet states

State	Multiplicity
Singlet	0.00 $kcal.mol^{-1}$
Tripltet	12.99 $kcal.mol^{-1}$

S3. Pairs determining Step (TDI – TDTS)

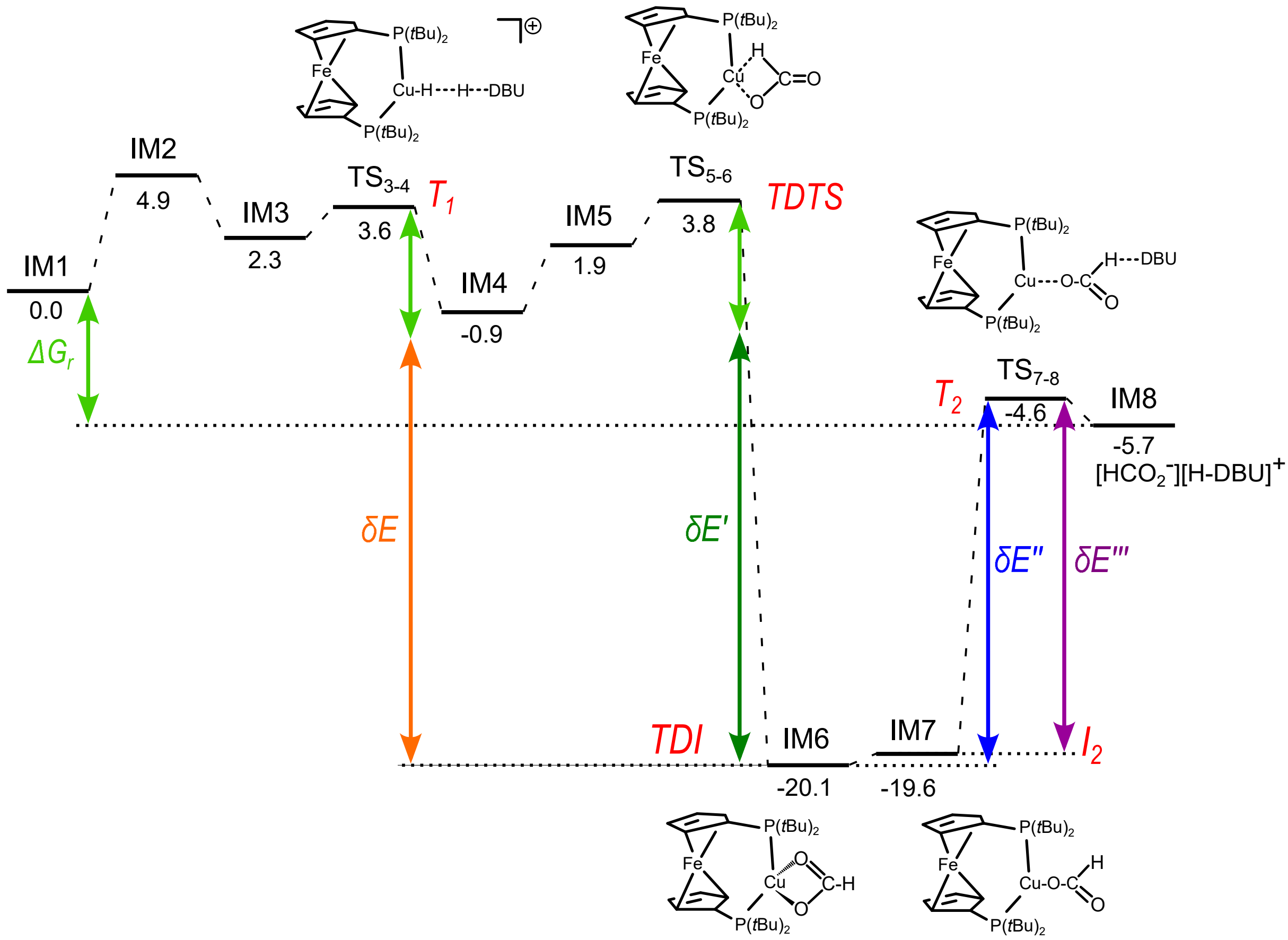

Fig. S2.: Assessment of potential TDTS and TDI Pairings. Relative free energies ΔG (in black) in kcal.mol^{-1} at 298.15K and 1atm

S4. Coordination Mode

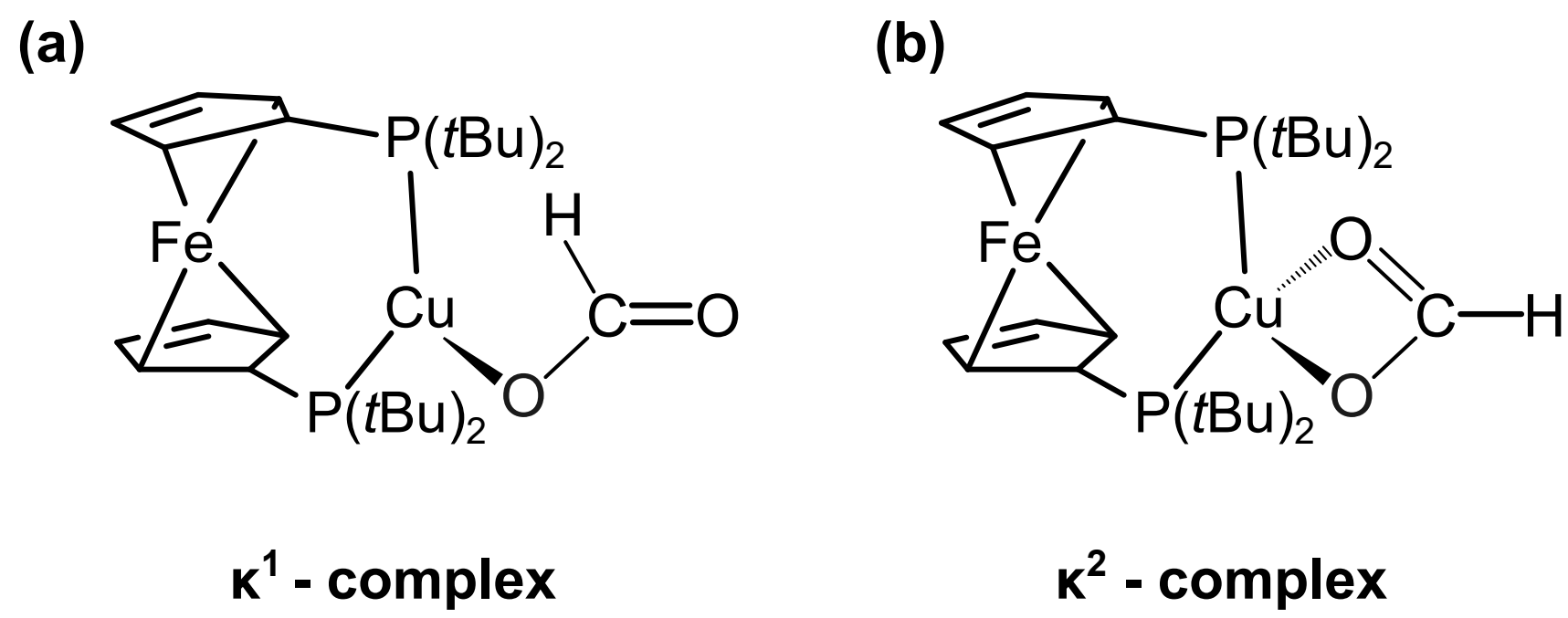

Fig. S3.: Eletronic structure of κ^1 and κ^2 isomers in DMF

S5. IRI and FBO Analysis

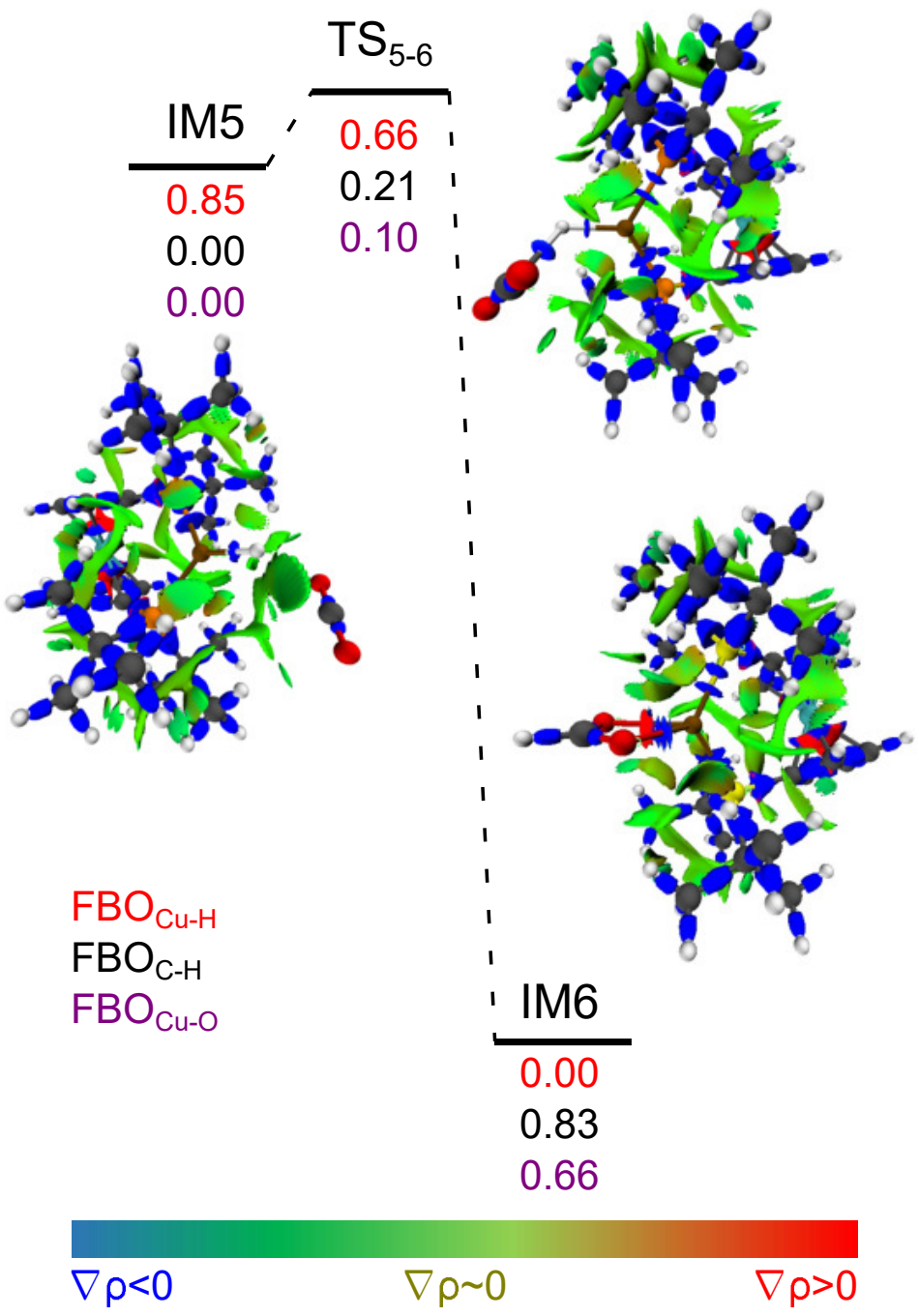

Fig. S4.: Analysis of IRI (Interaction Region Indicator) and FBO (Fuzzy Bond Order) of the interactions involved in formate formation.

S6. Bite Angle Analysis

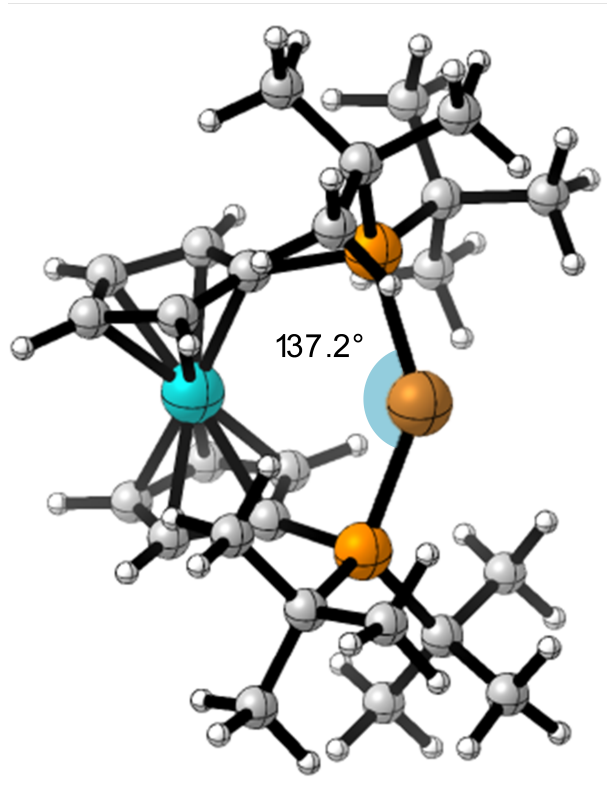

Table S2.: Influence of key bond distances and bite angle on the stability of intermediates and transition states in the catalytic cycle

**System**	$\Delta\theta_{\mathbf{P1-Cu2-P3}}/^{\circ}$	$\Delta\mathbf{d}_{\mathbf{P1-Cu2}}/\mathbf{\AA}$	$\Delta\mathbf{d}_{\mathbf{Cu2-P2}}/\mathbf{\AA}$
IM1	0.0	0.000	0.000
IM2	-11.9	0.032	0.034
IM3	-11.9	0.045	0.038
TS_{3-4}	-14.9	0.083	0.046
IM4	-23.9	0.083	0.085
IM5	-25.3	0.080	0.086
TS_{5-6}	-23.3	0.063	0.071
IM6	-16.3	0.025	0.025
IM7	-16.3	0.020	0.032
TS_{7-8}	0.5	-0.009	-0.009
IM1···DBU[a]	0.6	-0.002	-0.002

[a]vdW complex.

S7. Cartesian Coordinates

H2

G: -1.175546

1 0.000000000 0.000000000 0.380574000

1 0.000000000 0.000000000 -0.380574000

CO2

G: -188.451882

6 0.000000000 0.000000000 0.000000000

8 0.000000000 0.000000000 -1.163127000

8 0.000000000 0.000000000 1.163127000

HCOO-

G: -189.147339

8 -1.130850000 -0.212080000 0.000000000

8 1.130950000 -0.211125000 0.000000000

6 0.000000000 0.318934000 0.000000000

1 -0.000805000 1.472035000 0.000000000

DBU

G: -461.581871

6 2.789088000 -0.001347000 0.364977000

6 2.000298000 1.310313000 0.302978000

6 0.901170000 1.320298000 -0.769073000

6 1.945771000 -1.262255000 0.587345000

7 -0.316608000 0.606331000 -0.391821000

6 0.895927000 -1.543257000 -0.507433000

6 -0.398847000 -0.768883000 -0.348557000

1 3.346864000 -0.118506000 -0.582928000

1 1.533940000 1.527178000 1.280502000

1 1.299824000 0.923183000 -1.718670000

1 1.436993000 -1.211762000 1.566772000

1 0.602768000 -2.600771000 -0.484168000

1 3.550335000 0.070232000 1.160574000

1 2.700370000 2.137889000 0.096098000

1 0.604949000 2.357755000 -0.982727000

1 2.626876000 -2.127911000 0.639812000

1 1.339212000 -1.355677000 -1.501667000

7 -1.478310000 -1.451236000 -0.144930000

6 -1.464907000 1.434897000 -0.018235000

6 -2.717335000 -0.709942000 0.029979000

6 -2.496607000 0.620465000 0.751535000

1 -3.435315000 -1.336337000 0.587015000

1 -3.185634000 -0.516228000 -0.957074000

1 -2.123550000 0.417266000 1.770153000

1 -3.431572000 1.194112000 0.849356000

1 -1.922345000 1.875246000 -0.925219000

1 -1.106647000 2.276929000 0.598328000

H-DBU

G: -462.050236

6 -2.816317000 0.015005000 0.373975000

6 -2.023962000 -1.295864000 0.368706000

6 -0.949741000 -1.371444000 -0.720753000

6 -1.975076000 1.282774000 0.551479000

7 0.287800000 -0.635775000 -0.390457000

6 -0.918429000 1.508873000 -0.551503000

6 0.330608000 0.690879000 -0.362255000

1 -3.374445000 0.093804000 -0.576403000

1 -1.546986000 -1.467747000 1.348978000

1 -1.340290000 -1.007969000 -1.684382000

1 -1.474544000 1.281027000 1.534763000

1 -0.625498000 2.567175000 -0.577443000

1 -3.573492000 -0.024782000 1.173712000

1 -2.721452000 -2.133514000 0.207234000

1 -0.646014000 -2.412734000 -0.884187000

1 -2.644835000 2.156485000 0.545770000

1 -1.345781000 1.280828000 -1.541695000

7 1.466378000 1.339618000 -0.131660000

6 1.488390000 -1.448114000 -0.108064000

6 2.753715000 0.689721000 0.111415000

6 2.497014000 -0.676645000 0.731706000

1 3.339857000 1.333973000 0.780366000

1 3.304478000 0.598867000 -0.839581000

1 2.107532000 -0.549676000 1.754283000

1 3.433367000 -1.247640000 0.797515000

1 1.926705000 -1.769005000 -1.067885000

1 1.159035000 -2.349958000 0.425802000

1 1.425905000 2.352848000 -0.110260000

[HCOO$^-$][DBU]

G: -650.726382

7 -5.496530000 7.744843000 3.354239000

6 -5.625402000 4.263037000 0.506008000

6 -6.285830000 5.645213000 0.413671000

6 -5.670269000 4.258847000 3.095508000

7 -5.672328000 6.679586000 1.244567000

6 -6.493006000 5.552970000 3.281034000

6 -5.867468000 6.756645000 2.605353000

1 -7.042834000 3.239617000 1.780457000

1 -4.534245000 4.393232000 0.409784000

1 -7.361561000 5.570656000 0.649753000

1 -6.560108000 5.816490000 4.344844000

1 -5.419686000 2.544242000 1.799969000

1 -5.960451000 3.663265000 -0.359480000

1 -6.233044000 6.007824000 -0.624971000

1 -7.527102000 5.393787000 2.925932000

6 -5.967495000 3.502888000 1.792909000

6 -4.675334000 7.527271000 0.577729000

6 -4.840527000 8.851014000 2.674302000

6 -3.882415000 8.338317000 1.596041000

1 -4.302419000 9.464766000 3.417179000

1 -5.593175000 9.521396000 2.206734000

1 -3.133681000 7.688017000 2.075772000

1 -3.349761000 9.159156000 1.089423000

1 -5.186524000 8.196495000 -0.140935000

1 -3.989180000 6.874473000 0.012883000

1 -5.888451000 3.587276000 3.943305000

1 -4.595102000 4.506221000 3.147852000

1 -0.703217000 5.899404000 1.226028000

6 -1.736299000 5.426817000 1.365702000

8 -2.298150000 5.060033000 0.308451000

8 -2.133471000 5.355606000 2.549653000

[HCOO$^-$][H-DBU]$^+$

G: -651.219714

6 1.880462000 -2.505338000 0.405332000

6 2.858532000 -1.326617000 0.394207000

6 2.578024000 -0.296279000 -0.705647000

6 0.405724000 -2.122343000 0.560677000

7 1.476603000 0.629407000 -0.392403000

6 -0.153758000 -1.218470000 -0.557808000

6 0.196139000 0.235056000 -0.384522000

1 1.998711000 -3.068033000 -0.538720000

1 2.855599000 -0.809003000 1.369352000

1 2.373829000 -0.799166000 -1.664443000

1 0.236832000 -1.632521000 1.535681000

1 -1.251195000 -1.303495000 -0.571613000

1 2.161577000 -3.199754000 1.214476000

1 3.880971000 -1.710902000 0.244510000

1 3.464283000 0.329905000 -0.871396000

1 -0.201434000 -3.041672000 0.572277000

1 0.214667000 -1.560104000 -1.540229000

7 -0.792809000 1.083735000 -0.191687000

6 1.851038000 2.020046000 -0.080917000

6 -0.585286000 2.508286000 0.033987000

6 0.759873000 2.723462000 0.715463000

1 -1.419579000 2.872670000 0.650289000

1 -0.625377000 3.048508000 -0.928280000

1 0.722557000 2.311335000 1.736937000

1 0.992162000 3.795001000 0.794208000

1 2.059361000 2.554062000 -1.024206000

1 2.787034000 1.993927000 0.495804000

1 -1.805175000 0.737867000 -0.112245000

8 -3.353647000 0.575823000 0.154675000

8 -3.356335000 -1.652024000 -0.212752000

6 -3.884799000 -0.569651000 0.092556000

1 -4.987218000 -0.589924000 0.350877000

IM1

G: -3158.302808

26 -3.427674000 -0.345933000 -0.415790000

6 -3.251712000 0.982587000 1.149683000

6 -3.384544000 -2.357355000 0.132716000

6 -4.626823000 0.723963000 0.892874000

6 -2.657139000 1.565915000 -0.028969000

6 -2.087566000 -1.958147000 -0.341516000

6 -4.336226000 -2.145841000 -0.907332000

6 -4.900505000 1.115357000 -0.449845000

6 -3.698982000 1.631455000 -1.017332000

6 -2.276848000 -1.482763000 -1.690959000

6 -3.650128000 -1.615191000 -2.038180000

1 -2.742283000 0.765835000 2.081312000

1 -3.624839000 -2.744027000 1.118746000

1 -5.328620000 0.267878000 1.588934000

1 -5.406713000 -2.329454000 -0.831089000

1 -5.851088000 1.011172000 -0.970415000

1 -3.605531000 1.995245000 -2.036294000

1 -1.507536000 -1.080661000 -2.339765000

1 -4.097499000 -1.320765000 -2.985927000

15 -0.425468000 -2.021353000 0.441641000

15 -0.900608000 2.109201000 -0.069297000

6 0.547837000 -3.262058000 -0.639640000

6 1.797092000 -3.760657000 0.106757000

6 -0.322333000 -4.453632000 -1.073330000

6 1.020676000 -2.477220000 -1.881610000

1 2.415713000 -2.929788000 0.479914000

1 2.414249000 -4.343990000 -0.595904000

1 1.548904000 -4.423184000 0.946577000

1 -1.195321000 -4.131466000 -1.659007000

1 -0.681161000 -5.041356000 -0.217022000

1 0.279682000 -5.123656000 -1.709747000

1 0.187778000 -2.147750000 -2.515769000

1 1.657717000 -3.131885000 -2.498768000

1 1.612272000 -1.591651000 -1.598980000

6 -0.619020000 -2.595131000 2.243560000

6 0.746737000 -2.447132000 2.949495000

6 -1.576435000 -1.597257000 2.926869000

6 -1.138961000 -4.034308000 2.383302000

1 1.481838000 -3.195121000 2.633531000

1 0.595602000 -2.573890000 4.033888000

1 1.176325000 -1.445644000 2.784548000

1 -2.582051000 -1.585161000 2.492983000

1 -1.168941000 -0.575487000 2.879691000

1 -1.669717000 -1.867862000 3.990968000

1 -2.092989000 -4.192246000 1.861380000

1 -1.300483000 -4.257212000 3.451083000

1 -0.416430000 -4.769715000 2.002638000

29 0.133252000 0.156564000 0.355624000

1 -1.974222000 5.145631000 2.023502000

6 -0.813035000 3.492847000 1.247528000

6 -0.681942000 2.782369000 2.611364000

6 0.445279000 4.353518000 1.042066000

6 -2.069321000 4.379686000 1.235677000

1 -1.572612000 2.195903000 2.870791000

1 -0.552568000 3.540749000 3.400923000

1 0.192134000 2.111809000 2.633407000

1 0.385128000 4.978772000 0.141260000

1 1.360154000 3.743180000 0.991220000

1 0.547862000 5.033591000 1.903378000

1 -2.201425000 4.901691000 0.277737000

1 -2.981608000 3.801190000 1.440254000

6 -0.489773000 2.719269000 -1.822224000

6 -1.272231000 3.974328000 -2.239804000

6 1.030016000 2.984419000 -1.900619000

6 -0.781678000 1.547285000 -2.782245000

1 -0.502040000 1.849069000 -3.804484000

1 -1.833713000 1.243009000 -2.797397000

1 -0.173997000 0.667307000 -2.519435000

1 -2.360276000 3.838341000 -2.167593000

1 -0.997511000 4.849034000 -1.633763000

1 -1.034008000 4.215635000 -3.289068000

1 1.607730000 2.128362000 -1.515673000

1 1.306097000 3.128174000 -2.957941000

1 1.341560000 3.883323000 -1.357969000

IM2

G: -3159.468654

26 -0.027701000 2.190713000 -0.076308000

6 1.072013000 1.477137000 1.504330000
6 -2.075069000 2.285075000 0.281122000
6 1.029062000 2.898495000 1.560572000
6 1.696730000 1.075518000 0.267984000
6 -1.752804000 1.082374000 -0.438515000
6 -1.659240000 3.406829000 -0.494528000
6 1.604881000 3.399593000 0.357364000
6 2.018412000 2.288706000 -0.434843000
6 -1.124937000 1.500491000 -1.667914000
6 -1.081419000 2.922573000 -1.703848000
1 0.683609000 0.812575000 2.266125000
1 -2.538041000 2.351205000 1.261359000
1 0.592954000 3.490002000 2.363660000
1 -1.741477000 4.449606000 -0.192309000
1 1.687370000 4.446390000 0.069459000
1 2.479097000 2.369658000 -1.414908000
1 -0.733957000 0.845775000 -2.437362000
1 -0.643288000 3.525352000 -2.497415000
15 -2.057612000 -0.687359000 -0.061725000
15 1.996672000 -0.689069000 -0.141129000
6 -3.297804000 -1.219923000 -1.423404000
6 -4.011476000 -2.522927000 -1.024883000
6 -4.330996000 -0.124380000 -1.734601000
6 -2.454657000 -1.513380000 -2.682693000
1 -3.300215000 -3.317229000 -0.750475000
1 -4.591727000 -2.883646000 -1.889937000
1 -4.719228000 -2.380300000 -0.197552000
1 -3.852561000 0.803402000 -2.079603000
1 -4.959693000 0.116197000 -0.866317000
1 -4.996131000 -0.477540000 -2.540514000
1 -1.964601000 -0.616628000 -3.082630000
1 -3.114174000 -1.902835000 -3.475623000
1 -1.680519000 -2.271607000 -2.482411000
6 -2.804456000 -0.817146000 1.685531000
6 -2.865518000 -2.310254000 2.075916000
6 -1.807921000 -0.152849000 2.656663000
6 -4.194486000 -0.175013000 1.815951000
1 -3.619077000 -2.876258000 1.518068000
1 -3.124477000 -2.383567000 3.144755000
1 -1.889086000 -2.800816000 1.933880000
1 -1.661517000 0.916510000 2.470760000
1 -0.826618000 -0.648660000 2.608313000
1 -2.185871000 -0.268240000 3.685453000
1 -4.202967000 0.880229000 1.509456000
1 -4.515778000 -0.219649000 2.869918000
1 -4.950040000 -0.707831000 1.222284000
29 -0.030866000 -1.737319000 -0.136268000
1 4.885728000 -0.559429000 2.399972000
6 3.205992000 -1.263804000 1.230687000
6 2.333951000 -1.596722000 2.460195000
6 3.929934000 -2.553812000 0.809144000
6 4.232094000 -0.178744000 1.597190000
1 1.828062000 -0.714491000 2.872573000
1 2.975811000 -2.004196000 3.258524000
1 1.569135000 -2.353277000 2.219958000
1 4.657268000 -2.385090000 0.003973000
1 3.226398000 -3.339701000 0.493828000
1 4.489731000 -2.940444000 1.676491000

1 4.872902000 0.093071000 0.746987000

1 3.747280000 0.735724000 1.967545000

6 2.772498000 -0.777257000 -1.878248000

6 4.158947000 -0.121097000 -1.973274000

6 2.855016000 -2.259727000 -2.303405000

6 1.780780000 -0.101442000 -2.847344000

1 2.170384000 -0.192885000 -3.874140000

1 1.622089000 0.962495000 -2.641998000

1 0.802778000 -0.606147000 -2.818801000

1 4.156026000 0.926147000 -1.640858000

1 4.909794000 -0.663230000 -1.381728000

1 4.495763000 -0.137869000 -3.023219000

1 1.883072000 -2.765027000 -2.186851000

1 3.128306000 -2.303571000 -3.370274000

1 3.608305000 -2.830567000 -1.750288000

1 -0.438020000 -3.565176000 -0.252692000

1 0.347930000 -3.571736000 -0.249334000

IM3

G: -3621.052206

26 3.426135000 -1.103854000 -0.589882000

6 2.724121000 -2.113346000 1.054902000

6 4.447798000 0.645402000 -0.124762000

6 3.991447000 -2.640768000 0.677153000

6 1.799426000 -2.290682000 -0.038384000

6 3.098701000 0.955679000 -0.516444000

6 5.094110000 -0.031228000 -1.200566000

6 3.880887000 -3.126778000 -0.658197000

6 2.541155000 -2.915449000 -1.099348000

6 2.938639000 0.440074000 -1.853675000

6 4.164206000 -0.149295000 -2.273657000

1 2.503668000 -1.641792000 2.006032000

1 4.912333000 0.861471000 0.832469000

1 4.892611000 -2.630288000 1.287889000

1 6.111497000 -0.418778000 -1.183294000

1 4.683984000 -3.554068000 -1.256596000

1 2.168803000 -3.171092000 -2.087191000

1 2.032800000 0.477631000 -2.445528000

1 4.338879000 -0.640658000 -3.229285000

15 1.760244000 1.877071000 0.344232000

15 0.035923000 -1.802188000 0.096756000

6 1.512044000 3.411312000 -0.780183000

6 0.716016000 4.492356000 -0.027105000

6 2.841746000 3.995660000 -1.286371000

6 0.671497000 2.947912000 -1.990117000

1 -0.203664000 4.095937000 0.429027000

1 0.420455000 5.276997000 -0.742658000

1 1.313769000 4.977645000 0.755774000

1 3.422823000 3.256513000 -1.856507000

1 3.472052000 4.373420000 -0.470509000

1 2.625987000 4.842561000 -1.959596000

1 1.233608000 2.288309000 -2.662155000

1 0.374176000 3.830572000 -2.579670000

1 -0.245586000 2.425298000 -1.682879000

6 2.407674000 2.355398000 2.075781000

6 1.259380000 3.012788000 2.872256000

6 2.738497000 1.029853000 2.795375000

6 3.627920000 3.289601000 2.063145000

1 1.024470000 4.027522000 2.533157000
1 1.561990000 3.083317000 3.929882000
1 0.339025000 2.411604000 2.824916000
1 3.528344000 0.450186000 2.305485000
1 1.843621000 0.391731000 2.870833000
1 3.070634000 1.254559000 3.821934000
1 4.458898000 2.902638000 1.458143000
1 3.995513000 3.417059000 3.095165000
1 3.369549000 4.288793000 1.685376000
29 0.011856000 0.420542000 0.638641000
1 -0.524236000 -5.034775000 2.119807000
6 -0.644418000 -2.981483000 1.444998000
6 -0.185277000 -2.410966000 2.803764000
6 -2.182016000 -2.955371000 1.422707000
6 -0.125253000 -4.420256000 1.294935000
1 0.901720000 -2.471772000 2.940929000
1 -0.646254000 -3.000546000 3.613330000
1 -0.495569000 -1.362298000 2.929809000
1 -2.598527000 -3.482575000 0.554655000
1 -2.573090000 -1.927333000 1.430845000
1 -2.561676000 -3.464102000 2.323892000
1 -0.442670000 -4.885178000 0.351891000
1 0.972674000 -4.463433000 1.346790000
6 -0.808389000 -2.086301000 -1.587113000
6 -0.836722000 -3.549387000 -2.054887000
6 -2.248367000 -1.535508000 -1.496094000
6 -0.056638000 -1.206553000 -2.605251000
1 -0.546785000 -1.300159000 -3.587852000
1 0.995839000 -1.486833000 -2.725244000
1 -0.102350000 -0.146756000 -2.310968000
1 0.163300000 -4.001072000 -2.106326000
1 -1.459035000 -4.174967000 -1.400073000
1 -1.277359000 -3.595737000 -3.064986000
1 -2.292796000 -0.588416000 -0.937168000
1 -2.628040000 -1.349206000 -2.513696000
1 -2.938057000 -2.239372000 -1.019004000
6 -6.840030000 -1.385264000 0.185749000
6 -7.028678000 -0.384779000 -0.957532000
6 -6.728346000 1.066331000 -0.561720000
6 -5.440757000 -1.395244000 0.808244000
7 -5.303047000 1.400633000 -0.511984000
6 -5.002909000 -0.066312000 1.454872000
6 -4.476184000 0.980416000 0.493496000
1 -7.577308000 -1.157715000 0.977832000
1 -6.387344000 -0.652808000 -1.815742000
1 -7.205213000 1.299936000 0.405603000
1 -4.190795000 -0.248097000 2.171032000
1 -7.080815000 -2.399429000 -0.175270000
1 -8.071793000 -0.438721000 -1.312355000
1 -7.183197000 1.749669000 -1.292620000
1 -5.842133000 0.355787000 2.035743000
7 -3.259967000 1.399658000 0.671285000
6 -4.805694000 2.266796000 -1.587477000
6 -2.747027000 2.425387000 -0.218041000
6 -3.283012000 2.269834000 -1.638733000
1 -1.645467000 2.372436000 -0.205726000
1 -3.001831000 3.431169000 0.170191000
1 -2.918050000 1.320259000 -2.061577000

1 -2.931885000 3.080187000 -2.295430000

1 -5.186273000 3.294306000 -1.438629000

1 -5.219917000 1.905658000 -2.543183000

1 -5.407853000 -2.175976000 1.585553000

1 -4.697211000 -1.688575000 0.050252000

1 -1.781862000 0.865336000 1.674484000

1 -1.091177000 0.784773000 2.090762000

TS_{3-4}

G: -3621.050880

26 3.488007000 -1.043624000 -0.676937000

6 2.852308000 -2.114925000 0.959472000

6 4.514219000 0.710789000 -0.223312000

6 4.110983000 -2.616110000 0.522214000

6 1.891337000 -2.257959000 -0.105830000

6 3.140039000 1.006717000 -0.523626000

6 5.102316000 0.072269000 -1.355000000

6 3.959305000 -3.057816000 -0.823680000

6 2.603796000 -2.842080000 -1.211369000

6 2.902594000 0.524349000 -1.861703000

6 4.109352000 -0.032898000 -2.372570000

1 2.662848000 -1.686391000 1.935399000

1 5.031702000 0.913134000 0.710071000

1 5.030710000 -2.620068000 1.104664000

1 6.124421000 -0.298921000 -1.412097000

1 4.743638000 -3.458726000 -1.463751000

1 2.204785000 -3.067462000 -2.195583000

1 1.955770000 0.559619000 -2.388713000

1 4.232915000 -0.497032000 -3.349609000

15 1.826928000 1.869592000 0.422356000

15 0.129384000 -1.759774000 0.080709000

6 1.589430000 3.493452000 -0.570222000

6 0.821792000 4.523724000 0.275785000

6 2.916143000 4.089785000 -1.067759000

6 0.703608000 3.131979000 -1.779777000

1 -0.107864000 4.102316000 0.689680000

1 0.543969000 5.374421000 -0.368169000

1 1.425276000 4.925738000 1.100369000

1 3.461891000 3.385678000 -1.712687000

1 3.579802000 4.385005000 -0.244144000

1 2.703281000 4.993374000 -1.664077000

1 1.205246000 2.457521000 -2.485351000

1 0.451569000 4.052167000 -2.332625000

1 -0.237639000 2.662759000 -1.458221000

6 2.480092000 2.215538000 2.181479000

6 1.306361000 2.746069000 3.034775000

6 2.879969000 0.856124000 2.787702000

6 3.661669000 3.196015000 2.240784000

1 0.995249000 3.760544000 2.762192000

1 1.621619000 2.771856000 4.090975000

1 0.428140000 2.085294000 2.958909000

1 3.703108000 0.366617000 2.255754000

1 2.020462000 0.169574000 2.799783000

1 3.195394000 1.010161000 3.832621000

1 4.506438000 2.880313000 1.612742000

1 4.024591000 3.261961000 3.280344000

1 3.369928000 4.209901000 1.933861000

29 0.001557000 0.483109000 0.549137000

1 -0.310963000 -4.845473000 2.357788000
6 -0.489732000 -2.852116000 1.530426000
6 -0.041067000 -2.146743000 2.828994000
6 -2.026938000 -2.884191000 1.533455000
6 0.073868000 -4.280910000 1.491461000
1 1.048547000 -2.143735000 2.956441000
1 -0.464705000 -2.681470000 3.695174000
1 -0.398368000 -1.104519000 2.864897000
1 -2.435332000 -3.500749000 0.722244000
1 -2.455890000 -1.874492000 1.459205000
1 -2.373456000 -3.324658000 2.482736000
1 -0.226067000 -4.820808000 0.582603000
1 1.171958000 -4.289346000 1.548024000
6 -0.756401000 -2.169019000 -1.557498000
6 -0.712793000 -3.653265000 -1.952205000
6 -2.224137000 -1.699780000 -1.463720000
6 -0.082460000 -1.286486000 -2.630035000
1 -0.589464000 -1.445655000 -3.595591000
1 0.982698000 -1.501415000 -2.769704000
1 -0.184430000 -0.220473000 -2.371258000
1 0.307026000 -4.057524000 -1.998627000
1 -1.290942000 -4.273356000 -1.252292000
1 -1.168400000 -3.775661000 -2.949370000
1 -2.314542000 -0.712919000 -0.987746000
1 -2.635689000 -1.623867000 -2.483447000
1 -2.861889000 -2.399197000 -0.912430000
1 -2.027418000 1.161166000 0.798638000
1 -1.360136000 1.154989000 1.383689000
6 -6.778323000 -1.209057000 1.239430000
6 -7.361790000 -0.486989000 0.021388000
6 -6.996135000 1.000776000 -0.050171000
6 -5.254046000 -1.130105000 1.374447000
7 -5.639821000 1.262650000 -0.541779000
6 -4.690699000 0.299106000 1.522704000
6 -4.526056000 1.028819000 0.205759000
1 -7.232852000 -0.781504000 2.152124000
1 -7.029522000 -0.972984000 -0.912854000
1 -7.139167000 1.475884000 0.934893000
1 -4.770354000 -1.619278000 0.511560000
1 -3.696549000 0.259731000 1.985858000
1 -7.082733000 -2.268839000 1.212927000
1 -8.461112000 -0.572251000 0.048602000
1 -7.680355000 1.519385000 -0.735770000
1 -4.951025000 -1.708015000 2.262248000
1 -5.332016000 0.883689000 2.204016000
7 -3.316389000 1.344403000 -0.156266000
6 -5.523287000 1.820158000 -1.893943000
6 -3.081763000 1.971388000 -1.446049000
6 -4.137435000 1.561642000 -2.469239000
1 -2.077949000 1.676787000 -1.793501000
1 -3.061326000 3.072196000 -1.336244000
1 -4.026424000 0.487542000 -2.691053000
1 -4.013231000 2.112073000 -3.413859000
1 -5.738036000 2.904530000 -1.869000000
1 -6.292521000 1.351492000 -2.527251000

IM4

G: -3621.058558

26 3.553875000 -0.996921000 -0.725216000
6 2.959356000 -2.101875000 0.900537000
6 4.537852000 0.762576000 -0.221002000
6 4.206526000 -2.594979000 0.420829000
6 1.970335000 -2.219574000 -0.141986000
6 3.158691000 1.038324000 -0.522334000
6 5.149654000 0.166073000 -1.363474000
6 4.019852000 -3.007090000 -0.930095000
6 2.654542000 -2.781344000 -1.277066000
6 2.945324000 0.587471000 -1.875323000
6 4.167143000 0.067360000 -2.391205000
1 2.796024000 -1.692373000 1.889666000
1 5.045063000 0.948569000 0.721011000
1 5.140973000 -2.612393000 0.979296000
1 6.180171000 -0.181242000 -1.421193000
1 4.786781000 -3.395490000 -1.598530000
1 2.230308000 -2.985773000 -2.255218000
1 2.005200000 0.617512000 -2.413705000
1 4.307463000 -0.366986000 -3.379694000
15 1.813031000 1.824058000 0.454500000
15 0.215069000 -1.715906000 0.108320000
6 1.503835000 3.448607000 -0.527814000
6 0.741773000 4.458531000 0.347207000
6 2.788461000 4.087851000 -1.078356000
6 0.576445000 3.061245000 -1.698638000
1 -0.152250000 4.006937000 0.804773000
1 0.407495000 5.297782000 -0.285413000
1 1.369188000 4.882700000 1.142479000
1 3.326369000 3.405995000 -1.752808000
1 3.478649000 4.393332000 -0.280144000
1 2.525876000 4.991095000 -1.655850000
1 1.066846000 2.405230000 -2.429143000
1 0.263656000 3.971647000 -2.236936000
1 -0.328632000 2.554653000 -1.330179000
6 2.518286000 2.199090000 2.192455000
6 1.368396000 2.716891000 3.085343000
6 2.950997000 0.846141000 2.792948000
6 3.685533000 3.198034000 2.214555000
1 1.043436000 3.730958000 2.827648000
1 1.716946000 2.740179000 4.131461000
1 0.492355000 2.051901000 3.028992000
1 3.767897000 0.365394000 2.243986000
1 2.100341000 0.148587000 2.823217000
1 3.288562000 1.005339000 3.830486000
1 4.514035000 2.900087000 1.557387000
1 4.082814000 3.271229000 3.241474000
1 3.364773000 4.206603000 1.918123000
29 -0.110975000 0.521436000 0.682381000
1 -0.129857000 -4.917563000 2.258575000
6 -0.353599000 -2.893294000 1.518035000
6 0.116721000 -2.239502000 2.835894000
6 -1.889649000 -2.949817000 1.558278000
6 0.225640000 -4.311955000 1.407062000
1 1.209148000 -2.232843000 2.938972000
1 -0.283793000 -2.812315000 3.689075000
1 -0.246485000 -1.202035000 2.919230000
1 -2.310567000 -3.528000000 0.724922000
1 -2.331594000 -1.942654000 1.549596000

1 -2.206860000 -3.446118000 2.490523000

1 -0.089555000 -4.818716000 0.484476000

1 1.324771000 -4.308425000 1.436301000

6 -0.698848000 -2.097156000 -1.528734000

6 -0.669599000 -3.571119000 -1.961128000

6 -2.164054000 -1.627188000 -1.407583000

6 -0.045693000 -1.190424000 -2.593771000

1 -0.582930000 -1.309976000 -3.549006000

1 1.011420000 -1.415173000 -2.771892000

1 -0.122894000 -0.132693000 -2.295457000

1 0.346419000 -3.983508000 -2.016489000

1 -1.252840000 -4.203300000 -1.276310000

1 -1.125876000 -3.667018000 -2.961306000

1 -2.225602000 -0.625146000 -0.962414000

1 -2.607900000 -1.582475000 -2.415866000

1 -2.786330000 -2.302318000 -0.810603000

1 -2.675083000 1.326508000 0.344658000

1 -1.470673000 1.116288000 1.272762000

6 -6.667874000 -1.320243000 1.350625000

6 -7.320472000 -0.732174000 0.096078000

6 -7.119996000 0.778687000 -0.056734000

6 -5.157153000 -1.094420000 1.463089000

7 -5.791510000 1.155246000 -0.573854000

6 -4.727026000 0.387233000 1.517763000

6 -4.687600000 1.049089000 0.165192000

1 -7.156430000 -0.883480000 2.240456000

1 -6.940694000 -1.231910000 -0.811880000

1 -7.297001000 1.294176000 0.900605000

1 -4.632787000 -1.591198000 0.629804000

1 -3.718821000 0.467454000 1.945977000

1 -6.870186000 -2.403128000 1.388926000

1 -8.404869000 -0.925657000 0.132559000

1 -7.848837000 1.189416000 -0.766756000

1 -4.794039000 -1.575879000 2.384166000

1 -5.401100000 0.956041000 2.179167000

7 -3.514286000 1.475820000 -0.269427000

6 -5.734513000 1.679484000 -1.950965000

6 -3.295278000 2.045014000 -1.593768000

6 -4.346631000 1.500557000 -2.551228000

1 -2.281269000 1.767467000 -1.913528000

1 -3.336553000 3.145807000 -1.539011000

1 -4.157025000 0.430262000 -2.730434000

1 -4.292386000 2.018037000 -3.519024000

1 -6.029973000 2.742384000 -1.939122000

1 -6.480876000 1.133771000 -2.545014000

IM5

G: -3347.472144

26 1.213260000 -1.434731000 0.234806000

6 1.601724000 -0.420316000 -1.504545000

6 -0.468735000 -2.640281000 0.103373000

6 2.384363000 -1.610720000 -1.461425000

6 1.952621000 0.414720000 -0.382589000

6 -0.810508000 -1.394436000 0.738086000

6 0.544224000 -3.289890000 0.871227000

6 3.213258000 -1.544168000 -0.302927000

6 2.953748000 -0.305516000 0.357289000

6 0.028986000 -1.299582000 1.906990000

6 0.845438000 -2.464133000 1.993340000

1 0.850679000 -0.193710000 -2.253350000

1 -0.882551000 -3.031605000 -0.821345000

1 2.326374000 -2.437883000 -2.168196000

1 1.020423000 -4.237261000 0.619764000

1 3.904747000 -2.313097000 0.040734000

1 3.431311000 0.014519000 1.279246000

1 0.051806000 -0.473510000 2.607983000

1 1.592294000 -2.663108000 2.761179000

15 -2.042757000 -0.086324000 0.310502000

15 1.187363000 2.073074000 -0.145817000

6 -3.218082000 -0.144491000 1.836624000

6 -4.571955000 0.495204000 1.489279000

6 -3.442195000 -1.559266000 2.390309000

6 -2.554443000 0.738919000 2.914952000

1 -4.450825000 1.486133000 1.024055000

1 -5.149061000 0.632906000 2.419889000

1 -5.175521000 -0.138112000 0.824677000

1 -2.504716000 -2.025460000 2.727288000

1 -3.909203000 -2.226563000 1.652100000

1 -4.116714000 -1.506943000 3.263293000

1 -1.614319000 0.316480000 3.292269000

1 -3.235307000 0.833599000 3.778556000

1 -2.347947000 1.751090000 2.530225000

6 -2.928265000 -0.679868000 -1.276628000

6 -3.940374000 0.386042000 -1.745549000

6 -1.843009000 -0.756980000 -2.369766000

6 -3.662623000 -2.021884000 -1.128329000

1 -4.772317000 0.531248000 -1.048161000

1 -4.380545000 0.037925000 -2.696765000

1 -3.490209000 1.370000000 -1.948755000

1 -1.093591000 -1.536127000 -2.189424000

1 -1.319966000 0.207952000 -2.464452000

1 -2.322427000 -0.975029000 -3.339396000

1 -3.021812000 -2.822181000 -0.734271000

1 -4.032672000 -2.349089000 -2.116030000

1 -4.536654000 -1.930104000 -0.467012000

29 -1.137969000 2.046358000 0.006305000

1 3.609154000 3.263007000 -2.926869000

6 1.857161000 3.029043000 -1.673245000

6 0.954593000 2.616564000 -2.855860000

6 1.688175000 4.540061000 -1.448265000

6 3.320527000 2.712485000 -2.013812000

1 1.170363000 1.600117000 -3.211831000

1 1.121347000 3.299838000 -3.706135000

1 -0.111406000 2.656055000 -2.584888000

1 2.398235000 4.932186000 -0.707316000

1 0.667308000 4.795665000 -1.129278000

1 1.880102000 5.069405000 -2.397447000

1 4.011014000 3.014385000 -1.213963000

1 3.474929000 1.641454000 -2.213763000

6 1.886827000 2.794520000 1.480778000

6 3.403523000 3.030108000 1.489790000

6 1.151616000 4.118410000 1.781810000

6 1.480399000 1.812881000 2.596954000

1 1.766183000 2.240042000 3.573316000

1 1.961140000 0.831768000 2.510609000

1 0.389471000 1.663413000 2.603850000

1 3.977186000 2.127597000 1.235781000

1 3.694759000 3.827586000 0.791232000

1 3.717470000 3.349136000 2.499495000

1 0.059554000 3.993858000 1.707385000

1 1.391157000 4.429857000 2.813091000

1 1.447293000 4.938958000 1.117673000

1 -2.092312000 3.349798000 0.076003000

6 -2.056566000 4.500235000 -2.596859000

8 -1.585703000 5.469060000 -2.158187000

8 -2.536443000 3.561042000 -3.089747000

TS_{5-6}

G: -3347.468370

26 1.234490000 -1.431987000 0.283000000

6 1.669940000 -0.462052000 -1.475161000

6 -0.437117000 -2.660389000 0.146657000

6 2.464128000 -1.640277000 -1.369577000

6 1.970813000 0.410948000 -0.367631000

6 -0.800468000 -1.408127000 0.753797000

6 0.572124000 -3.283252000 0.941013000

6 3.252054000 -1.528617000 -0.186711000

6 2.955034000 -0.274557000 0.426463000

6 0.020247000 -1.279673000 1.932661000

6 0.847523000 -2.433676000 2.052364000

1 0.950022000 -0.265812000 -2.261437000

1 -0.837850000 -3.072766000 -0.775063000

1 2.440440000 -2.488009000 -2.053542000

1 1.063336000 -4.229341000 0.714856000

1 3.940667000 -2.277607000 0.203536000

1 3.398463000 0.077751000 1.353379000

1 0.022930000 -0.438826000 2.617044000

1 1.585434000 -2.610289000 2.834217000

15 -2.033195000 -0.120605000 0.287848000

15 1.182451000 2.068341000 -0.192157000

6 -3.264429000 -0.188350000 1.761767000

6 -4.605779000 0.446125000 1.360553000

6 -3.499190000 -1.607919000 2.298059000

6 -2.647722000 0.694225000 2.867489000

1 -4.470079000 1.443841000 0.914409000

1 -5.226009000 0.567801000 2.265205000

1 -5.173125000 -0.180930000 0.659172000

1 -2.571884000 -2.066390000 2.672397000

1 -3.926243000 -2.275442000 1.535812000

1 -4.212099000 -1.567524000 3.140591000

1 -1.717478000 0.277696000 3.275875000

1 -3.359575000 0.779384000 3.706717000

1 -2.435439000 1.709742000 2.494175000

6 -2.854515000 -0.679407000 -1.343378000

6 -3.787289000 0.450434000 -1.830830000

6 -1.720118000 -0.776449000 -2.383145000

6 -3.632785000 -1.999346000 -1.257710000

1 -4.698700000 0.548453000 -1.230072000

1 -4.102522000 0.224297000 -2.864151000

1 -3.272286000 1.423963000 -1.839751000

1 -0.997062000 -1.571225000 -2.168041000

1 -1.174292000 0.177992000 -2.450295000

1 -2.158086000 -0.982959000 -3.374526000

1 -3.019502000 -2.831839000 -0.884891000

1 -3.993603000 -2.277514000 -2.263815000

1 -4.515628000 -1.908696000 -0.608434000

29 -1.123775000 1.991247000 -0.030192000

1 3.606088000 3.189273000 -2.991674000

6 1.844436000 2.989195000 -1.744409000

6 0.913009000 2.581424000 -2.904941000

6 1.713912000 4.509566000 -1.558268000

6 3.296808000 2.629553000 -2.091281000

1 0.984681000 1.514154000 -3.152866000

1 1.193050000 3.144418000 -3.812292000

1 -0.138881000 2.810844000 -2.673692000

1 2.428181000 4.906063000 -0.823632000

1 0.696773000 4.802682000 -1.257585000

1 1.927940000 5.004956000 -2.521095000

1 3.995107000 2.890280000 -1.283165000

1 3.415469000 1.558742000 -2.313428000

6 1.850461000 2.843530000 1.421547000

6 3.364240000 3.096066000 1.442343000

6 1.094880000 4.166299000 1.671830000

6 1.435831000 1.892109000 2.561547000

1 1.695085000 2.355911000 3.528512000

1 1.931231000 0.915742000 2.518643000

1 0.346939000 1.726316000 2.550871000

1 3.950163000 2.189858000 1.233646000

1 3.657684000 3.866845000 0.715141000

1 3.660597000 3.459401000 2.442290000

1 0.004692000 4.018983000 1.610004000

1 1.332726000 4.522685000 2.688734000

1 1.372061000 4.964514000 0.973866000

1 -2.149358000 3.263325000 -0.080734000

6 -2.165691000 4.556597000 -1.529004000

8 -1.821644000 5.525720000 -0.951767000

8 -2.545042000 3.904300000 -2.437186000

IM6

G: -3347.493583

26 -0.000087000 -2.552624000 0.000079000

6 -1.143508000 -1.866838000 1.560477000

6 2.033602000 -2.606848000 0.429675000

6 -1.118353000 -3.291177000 1.576466000

6 -1.726861000 -1.418830000 0.320409000

6 1.726784000 -1.419016000 -0.320281000

6 1.658941000 -3.748953000 -0.339608000

6 -1.659240000 -3.748773000 0.339784000

6 -2.033770000 -2.606647000 -0.429532000

6 1.143401000 -1.866991000 -1.560344000

6 1.118091000 -3.291328000 -1.576296000

1 -0.765015000 -1.229942000 2.350816000

1 2.459718000 -2.650369000 1.427660000

1 -0.716210000 -3.912120000 2.375351000

1 1.744659000 -4.785611000 -0.017194000

1 -1.745066000 -4.785430000 0.017393000

1 -2.459877000 -2.650149000 -1.427522000

1 0.764972000 -1.230066000 -2.350691000

1 0.715876000 -3.912246000 -2.375165000

15 1.976383000 0.368103000 0.046093000

15 -1.976238000 0.368311000 -0.046058000

6 3.220883000 0.901825000 -1.314419000

6 3.861918000 2.245732000 -0.927591000
6 4.307635000 -0.147115000 -1.595133000
6 2.382846000 1.143962000 -2.589549000
1 3.099034000 2.989921000 -0.651705000
1 4.417080000 2.638757000 -1.795859000
1 4.579606000 2.145785000 -0.102476000
1 3.875015000 -1.105012000 -1.919352000
1 4.940534000 -0.337962000 -0.717259000
1 4.962824000 0.215487000 -2.406156000
1 1.961649000 0.217037000 -2.999341000
1 3.033037000 1.573005000 -3.370910000
1 1.562015000 1.850253000 -2.392827000
6 2.738849000 0.498666000 1.792252000
6 2.835924000 1.995056000 2.168199000
6 1.700999000 -0.119829000 2.752412000
6 4.109012000 -0.174758000 1.957464000
1 3.666616000 2.507924000 1.670442000
1 3.010456000 2.074009000 3.254511000
1 1.903436000 2.529742000 1.928888000
1 1.531384000 -1.189007000 2.582620000
1 0.734860000 0.400138000 2.655896000
1 2.050000000 0.006741000 3.790645000
1 4.103759000 -1.232220000 1.658917000
1 4.415032000 -0.127036000 3.016739000
1 4.885271000 0.337966000 1.371525000
29 0.000143000 1.494219000 -0.000121000
1 -4.963012000 0.216727000 2.405821000
6 -3.220630000 0.902415000 1.314381000
6 -2.382641000 1.144336000 2.589590000
6 -3.861184000 2.246502000 0.927373000
6 -4.307777000 -0.146152000 1.594960000
1 -1.961794000 0.217297000 2.999477000
1 -3.032775000 1.573627000 3.370863000
1 -1.561558000 1.850349000 2.392926000
1 -4.578544000 2.146766000 0.101940000
1 -3.098004000 2.990518000 0.651841000
1 -4.416612000 2.639620000 1.795427000
1 -4.940558000 -0.336850000 0.716962000
1 -3.875542000 -1.104167000 1.919337000
6 -2.738908000 0.498861000 -1.792105000
6 -4.109372000 -0.174049000 -1.956920000
6 -2.835531000 1.995244000 -2.168184000
6 -1.701456000 -0.120189000 -2.752345000
1 -2.050598000 0.006330000 -3.790537000
1 -1.532221000 -1.189405000 -2.582395000
1 -0.735099000 0.399423000 -2.656095000
1 -4.104468000 -1.231483000 -1.658276000
1 -4.885278000 0.339035000 -1.370821000
1 -4.415646000 -0.126301000 -3.016120000
1 -1.902721000 2.529572000 -1.929329000
1 -3.010497000 2.074155000 -3.254428000
1 -3.665808000 2.508494000 -1.670112000
1 0.000283000 5.179159000 -0.000390000
6 0.000225000 4.059771000 -0.000247000
8 0.179062000 3.471339000 -1.097756000
8 -0.178674000 3.471621000 1.097393000

IM7

G: -3809.062835

29 -2.165912000 1.691980000 0.803358000

6 0.858580000 0.642533000 -1.283565000

6 -0.225697000 -2.377840000 0.001365000

6 1.950068000 -0.236732000 -1.536205000

6 1.096953000 1.345747000 -0.047418000

6 -0.841591000 -1.437601000 0.898770000

6 1.033155000 -2.775334000 0.542111000

6 2.867749000 -0.112441000 -0.452842000

6 2.350951000 0.856669000 0.457663000

6 0.080223000 -1.265362000 1.993870000

6 1.217952000 -2.094919000 1.780464000

1 -0.014678000 0.747971000 -1.916283000

1 -0.624353000 -2.726503000 -0.946500000

1 2.040443000 -0.911875000 -2.385555000

1 1.738858000 -3.455946000 0.068229000

1 3.788932000 -0.677790000 -0.320003000

1 2.833796000 1.147837000 1.385841000

1 -0.054609000 -0.604192000 2.841004000

1 2.088550000 -2.160655000 2.430934000

15 -2.457579000 -0.558825000 0.834970000

15 -0.083732000 2.600469000 0.590384000

6 -3.372773000 -1.216977000 2.385982000

6 -4.881355000 -0.944006000 2.258920000

6 -3.123764000 -2.710430000 2.648421000

6 -2.850762000 -0.380732000 3.574948000

1 -5.086123000 0.105800000 1.996952000

1 -5.361986000 -1.146822000 3.230385000

1 -5.363455000 -1.593922000 1.516594000

1 -2.056209000 -2.925861000 2.800836000

1 -3.485417000 -3.342106000 1.824919000

1 -3.660955000 -3.013965000 3.563383000

1 -1.793106000 -0.572655000 3.795812000

1 -3.422497000 -0.640848000 4.481391000

1 -2.973954000 0.698945000 3.389569000

6 -3.331455000 -1.068768000 -0.783038000

6 -4.626245000 -0.235211000 -0.922970000

6 -2.404015000 -0.615346000 -1.930677000

6 -3.652775000 -2.566460000 -0.894594000

1 -5.433202000 -0.583101000 -0.268686000

1 -4.989092000 -0.323465000 -1.960547000

1 -4.445525000 0.830935000 -0.711769000

1 -1.429591000 -1.115733000 -1.933340000

1 -2.230386000 0.470846000 -1.875686000

1 -2.896102000 -0.827015000 -2.894153000

1 -2.766347000 -3.202970000 -0.766034000

1 -4.074374000 -2.776333000 -1.892450000

1 -4.402486000 -2.877019000 -0.152773000

26 1.010403000 -0.722717000 0.243352000

1 1.340343000 5.008982000 -2.076285000

6 -0.025139000 3.976274000 -0.747299000

6 -0.951224000 3.502145000 -1.889312000

6 -0.629004000 5.274056000 -0.183787000

6 1.387387000 4.231517000 -1.294366000

1 -0.568677000 2.609784000 -2.401082000

1 -1.026814000 4.300620000 -2.646518000

1 -1.964807000 3.287042000 -1.515612000

1 0.023868000 5.756707000 0.555757000

1 -1.614973000 5.095756000 0.271318000

1 -0.769379000 5.991481000 -1.009242000

1 2.079046000 4.583785000 -0.516603000

1 1.816365000 3.326409000 -1.749001000

6 0.566763000 3.222896000 2.271867000

6 1.911224000 3.962802000 2.205630000

6 -0.511981000 4.137924000 2.891445000

6 0.665806000 1.991322000 3.194580000

1 0.961680000 2.323339000 4.203341000

1 1.396134000 1.247799000 2.857638000

1 -0.313304000 1.496429000 3.280155000

1 2.700737000 3.364020000 1.729978000

1 1.827964000 4.911304000 1.656468000

1 2.245587000 4.206107000 3.228567000

1 -1.502095000 3.655428000 2.878092000

1 -0.248286000 4.340790000 3.942628000

1 -0.601239000 5.103165000 2.384608000

7 -4.883462000 8.987399000 2.981970000

6 -0.547283000 9.002443000 4.037026000

6 -1.564262000 7.860910000 4.176201000

6 -2.308209000 10.852991000 4.463772000

7 -2.752287000 8.007320000 3.340066000

6 -3.503189000 9.912574000 4.723706000

6 -3.759742000 8.907478000 3.616347000

1 -0.919224000 10.032164000 5.891474000

1 -0.424220000 9.228795000 2.962769000

1 -1.853999000 7.743674000 5.234425000

1 -4.429649000 10.495952000 4.804720000

1 -0.161976000 11.039204000 4.654869000

1 0.433292000 8.648090000 4.398926000

1 -1.092199000 6.909694000 3.891528000

1 -3.364812000 9.397393000 5.691021000

6 -0.934351000 10.266641000 4.810616000

6 -2.770976000 7.213002000 2.109120000

6 -5.103789000 8.025073000 1.911828000

6 -3.822618000 7.721482000 1.133872000

1 -5.885152000 8.412682000 1.235404000

1 -5.490462000 7.068119000 2.314264000

1 -3.467476000 8.642547000 0.639996000

1 -4.001487000 6.956347000 0.364799000

1 -2.968103000 6.152935000 2.349120000

1 -1.769421000 7.257645000 1.648261000

1 -2.453415000 11.766959000 5.063637000

1 -2.328769000 11.173514000 3.406590000

1 -3.877967000 3.789161000 2.417460000

6 -4.249789000 3.834524000 1.353655000

8 -5.094726000 4.688840000 1.065168000

8 -3.728064000 2.969699000 0.568661000

TS_{7-8}

G: -3809.035284

29 -1.810186000 1.610243000 0.857096000

6 0.602338000 0.413135000 -1.505381000

6 -0.333634000 -2.423031000 0.315491000

6 1.605450000 -0.557129000 -1.783343000

6 1.050008000 1.254077000 -0.421815000

6 -0.834508000 -1.364299000 1.148399000

6 0.987718000 -2.751209000 0.739134000

6 2.674437000 -0.353594000 -0.863042000

6 2.338509000 0.753126000 -0.028890000

6 0.221287000 -1.043894000 2.078185000

6 1.326915000 -1.906167000 1.835768000

1 -0.344285000 0.494074000 -2.026743000

1 -0.851747000 -2.898088000 -0.512455000

1 1.542145000 -1.339209000 -2.537978000

1 1.632295000 -3.497618000 0.277669000

1 3.578800000 -0.954589000 -0.782274000

1 2.961823000 1.128581000 0.777299000

1 0.189675000 -0.267672000 2.834097000

1 2.276238000 -1.888057000 2.368283000

15 -2.460024000 -0.506827000 1.198936000

15 0.051120000 2.678094000 0.184543000

6 -3.177825000 -0.975726000 2.907612000

6 -4.692306000 -0.708761000 2.944015000

6 -2.887092000 -2.440502000 3.274490000

6 -2.507163000 -0.034017000 3.930350000

1 -4.938284000 0.318063000 2.632348000

1 -5.045189000 -0.834389000 3.980698000

1 -5.258509000 -1.414338000 2.321460000

1 -1.807637000 -2.647215000 3.309140000

1 -3.349234000 -3.146283000 2.570143000

1 -3.300609000 -2.648427000 4.275668000

1 -1.429171000 -0.215502000 4.027903000

1 -2.954477000 -0.204745000 4.923469000

1 -2.658445000 1.024434000 3.664731000

6 -3.523612000 -1.121304000 -0.251347000

6 -4.797028000 -0.249566000 -0.320510000

6 -2.724587000 -0.833172000 -1.539194000

6 -3.897388000 -2.608855000 -0.160985000

1 -5.514938000 -0.466398000 0.477862000

1 -5.304173000 -0.445337000 -1.279378000

1 -4.549641000 0.823773000 -0.282464000

1 -1.769780000 -1.367440000 -1.592486000

1 -2.521248000 0.244741000 -1.635994000

1 -3.331523000 -1.135017000 -2.408100000

1 -3.017464000 -3.261848000 -0.076433000

1 -4.444306000 -2.900180000 -1.073168000

1 -4.556891000 -2.813898000 0.693987000

26 0.917326000 -0.760238000 0.160054000

1 1.130619000 4.559829000 -2.984538000

6 -0.060261000 3.844195000 -1.326542000

6 -1.191748000 3.287257000 -2.217035000

6 -0.468893000 5.259000000 -0.879306000

6 1.257237000 3.889564000 -2.117642000

1 -0.950300000 2.305958000 -2.645955000

1 -1.357417000 3.977122000 -3.061036000

1 -2.136475000 3.199572000 -1.657317000

1 0.331144000 5.766875000 -0.323696000

1 -1.390585000 5.254871000 -0.276420000

1 -0.664668000 5.861758000 -1.782283000

1 2.090108000 4.278837000 -1.515222000

1 1.543516000 2.899023000 -2.500015000

6 0.950013000 3.460631000 1.665339000

6 2.319544000 4.061166000 1.311578000

6 0.036542000 4.543085000 2.281493000

6 1.092399000 2.349216000 2.725969000

1 1.566660000 2.774563000 3.625262000

1 1.699165000 1.499208000 2.394922000

1 0.101636000 1.970008000 3.021664000

1 2.995945000 3.334920000 0.839738000

1 2.222916000 4.923930000 0.637318000

1 2.804867000 4.420519000 2.234541000

1 -0.995826000 4.189129000 2.433971000

1 0.450945000 4.823782000 3.264251000

1 -0.011352000 5.456371000 1.679230000

7 -4.429754000 9.216713000 1.993551000

6 -1.607120000 7.978026000 5.260363000

6 -2.113843000 7.185996000 4.048433000

6 -3.758149000 9.395513000 5.465873000

7 -2.620618000 8.011035000 2.954269000

6 -4.545898000 8.699936000 4.338621000

6 -3.843451000 8.643369000 2.995215000

1 -3.247541000 7.648092000 6.616409000

1 -0.979376000 8.810193000 4.894419000

1 -2.873721000 6.447072000 4.352683000

1 -5.484911000 9.235825000 4.148317000

1 -2.273351000 9.069031000 7.005339000

1 -0.947272000 7.322317000 5.854770000

1 -1.289826000 6.591681000 3.632423000

1 -4.822328000 7.678664000 4.657691000

6 -2.721176000 8.510203000 6.165595000

6 -1.787458000 8.064276000 1.750581000

6 -3.774480000 9.100681000 0.697646000

6 -2.250687000 9.175444000 0.817869000

1 -4.153159000 9.892642000 0.028513000

1 -4.040289000 8.129026000 0.236737000

1 -1.966214000 10.160987000 1.226305000

1 -1.757835000 9.067872000 -0.161801000

1 -1.842219000 7.093202000 1.230245000

1 -0.738999000 8.227138000 2.055828000

1 -4.479680000 9.746537000 6.222620000

1 -3.272913000 10.301829000 5.060844000

1 -4.692808000 5.932118000 2.205690000

6 -3.918793000 5.217415000 1.783910000

8 -3.570961000 5.437188000 0.599872000

8 -3.524076000 4.322533000 2.570651000

IM1···DBU

G: -3620.70005401

29 -1.036441000 1.743126000 0.348978000

6 1.245748000 -0.129880000 -1.627839000

6 -0.553154000 -2.545274000 0.142447000

6 2.000454000 -1.329271000 -1.753859000

6 1.766770000 0.637413000 -0.522192000

6 -0.870370000 -1.350210000 0.875332000

6 0.588731000 -3.156752000 0.737858000

6 2.978930000 -1.339178000 -0.717806000

6 2.839408000 -0.138547000 0.038235000

6 0.117039000 -1.240966000 1.922086000

6 0.996698000 -2.356406000 1.844273000

1 0.412059000 0.149207000 -2.261848000

1 -1.072621000 -2.928019000 -0.731000000

1 1.827060000 -2.115363000 -2.486678000

1 1.079043000 -4.061230000 0.381525000

1 3.690563000 -2.136901000 -0.511145000
1 3.444454000 0.118377000 0.902634000
1 0.189951000 -0.441478000 2.650459000
1 1.853549000 -2.536578000 2.491096000
15 -2.232429000 -0.129083000 0.692339000
15 1.097891000 2.295916000 -0.089866000
6 -3.225901000 -0.303149000 2.318261000
6 -4.639474000 0.282081000 2.156056000
6 -3.315167000 -1.766896000 2.782107000
6 -2.479764000 0.538848000 3.375193000
1 -4.623051000 1.310733000 1.765946000
1 -5.121178000 0.311580000 3.146869000
1 -5.275468000 -0.331353000 1.504145000
1 -2.322400000 -2.198065000 2.974131000
1 -3.830699000 -2.404501000 2.050335000
1 -3.886958000 -1.808997000 3.724251000
1 -1.484609000 0.138259000 3.607315000
1 -3.058142000 0.532612000 4.313612000
1 -2.363123000 1.585424000 3.052664000
6 -3.247510000 -0.576583000 -0.851178000
6 -4.275680000 0.547341000 -1.106335000
6 -2.267094000 -0.554487000 -2.042336000
6 -3.959978000 -1.934517000 -0.751397000
1 -5.102343000 0.550905000 -0.388042000
1 -4.712421000 0.399819000 -2.107431000
1 -3.799937000 1.540242000 -1.092251000
1 -1.463318000 -1.294589000 -1.963456000
1 -1.807526000 0.440965000 -2.147232000
1 -2.828140000 -0.760182000 -2.968078000
1 -3.269088000 -2.762606000 -0.541383000
1 -4.459551000 -2.150504000 -1.710394000
1 -4.735720000 -1.932971000 0.027157000
26 1.074552000 -1.244000000 0.098036000
1 2.987173000 3.658690000 -3.141622000
6 1.465272000 3.361871000 -1.634392000
6 0.340828000 3.058312000 -2.647092000
6 1.384703000 4.856831000 -1.283326000
6 2.833830000 3.027601000 -2.250213000
1 0.348416000 2.016214000 -2.991300000
1 0.480017000 3.694482000 -3.536654000 1
-0.650713000 3.280964000 -2.223442000
1 2.214791000 5.185004000 -0.643648000
1 0.426264000 5.112923000 -0.808115000
1 1.449713000 5.437502000 -2.218391000
1 3.662375000 3.225720000 -1.555859000
1 2.896541000 1.977328000 -2.569607000
6 1.988054000 2.913105000 1.473768000
6 3.496139000 3.135129000 1.276422000
6 1.322398000 4.224069000 1.943354000
6 1.722999000 1.863644000 2.572887000
1 2.179735000 2.210899000 3.513573000
1 2.134448000 0.874788000 2.343235000
1 0.641624000 1.752860000 2.750095000
1 4.014065000 2.240574000 0.904862000
1 3.698009000 3.959359000 0.578037000
1 3.949905000 3.407518000 2.243828000
1 0.227600000 4.131627000 2.002005000
1 1.691375000 4.463172000 2.953823000

1 1.557549000 5.078281000 1.301476000

7 -2.137793000 5.578244000 -0.593835000

6 -0.966181000 8.034808000 2.936775000

6 -2.372541000 7.790223000 2.372668000

6 0.188217000 7.920581000 0.628362000

7 -2.484049000 6.607783000 1.518347000

6 -1.093049000 7.708174000 -0.202209000

6 -1.965760000 6.550438000 0.245497000

1 -0.387702000 9.700076000 1.699412000

1 -0.559521000 7.069753000 3.288132000

1 -2.722775000 8.687716000 1.833712000

1 -0.829177000 7.487485000 -1.244234000

1 0.974080000 8.837827000 2.420417000

1 -1.054590000 8.683977000 3.824496000

1 -3.080312000 7.645586000 3.201205000

1 -1.682651000 8.642195000 -0.214854000

6 -0.007309000 8.689557000 1.938590000

6 -3.232913000 5.476953000 2.067993000

6 -2.962539000 4.456340000 -0.184875000

6 -2.904308000 4.194382000 1.319110000

1 -2.637737000 3.557514000 -0.742863000

1 -4.014839000 4.623260000 -0.489361000

1 -1.882998000 3.871700000 1.590676000

1 -3.592649000 3.391368000 1.619180000

1 -4.319702000 5.680102000 2.016314000

1 -2.976832000 5.369670000 3.134707000

1 0.910851000 8.472277000 0.004800000

1 0.649175000 6.940997000 0.833983000
